# The presence and prognosis of nerve pathology following whiplash injury: a prospective cohort study

**DOI:** 10.1093/brain/awaf088

**Published:** 2025-03-05

**Authors:** Joel Fundaun, Colette Ridehalgh, Soraya Koushesh, Alex Novak, Macarena Tejos-Bravo, Stephen Bremner, Georgios Baskozos, Andrew Dilley, Annina B Schmid

**Affiliations:** Nuffield Department of Clinical Neurosciences, University of Oxford, Oxford OX3 9DU, UK; Department of Anesthesiology, Perioperative and Pain Medicine, Stanford University School of Medicine, Palo Alto, CA 94304, USA; Department of Clinical Neuroscience, Brighton and Sussex Medical School, University of Sussex, Brighton BN1 9PX, UK; School of Life Course and Population Sciences, Faculty of Life Sciences and Medicine, King’s College London, London SE1 1UL, UK; Nuffield Department of Clinical Neurosciences, University of Oxford, Oxford OX3 9DU, UK; Emergency Medicine Research Oxford (EMROx), Oxford University Hospitals NHS Foundation Trust, Oxford OX3 9DU, UK; Nuffield Department of Clinical Neurosciences, University of Oxford, Oxford OX3 9DU, UK; Departamento Fisiología, Pontificia Universidad Católica de Chile, Santiago, Chile; Department of Primary Care & Public Health, Brighton and Sussex Medical School, University of Brighton Falmer Campus, Brighton BN1 9PH, UK; Nuffield Department of Clinical Neurosciences, University of Oxford, Oxford OX3 9DU, UK; Department of Clinical Neuroscience, Brighton and Sussex Medical School, University of Sussex, Brighton BN1 9PX, UK; Nuffield Department of Clinical Neurosciences, University of Oxford, Oxford OX3 9DU, UK

**Keywords:** whiplash-associated disorders, motor vehicle collision, neuropathic pain, nerve injury, neuropathy

## Abstract

Whiplash-associated disorders (WADs) affect 20–50 million individuals globally each year, with ≤50% developing persistent pain. WAD grade II (WADII) is the most common type and is characterized by neck symptoms and musculoskeletal signs without apparent nerve injury on routine diagnostic testing. However, emerging evidence suggests that nerve pathology might be present in some people with WADII. This longitudinal cohort study aimed to investigate comprehensively the presence, temporal patterns and prognostic value of nerve pathology and neuropathic pain in acute WADII.

A prospective longitudinal cohort study was conducted with 129 acute participants with WADII (median age 36.0 years, 58% female) and 36 healthy controls (median age 39.0 years, 61% female). Participants with WADII were recruited within 4 weeks of injury from local emergency departments. Data collection included bedside neurological assessments, quantitative sensory testing, intraepidermal nerve fibre density and serum neurofilament light chain concentrations. Follow-up assessments were conducted 6 months after injury.

Signs of neuropathic pain were present in 65% (84/129) acutely and persisted in 32% (21/66) 6 months post-injury. Bedside neurological assessment revealed that somatosensory loss-of-function was present in 54% (70/129) acutely, reducing to 25% (17/67) 6 months post-injury. Quantitative sensory testing demonstrated significantly reduced cold, warm, thermal sensory limen, mechanical and vibration detection thresholds in acute WADII compared with controls (*d* > 0.47). Acute loss-of-function in at least one quantitative sensory testing parameter was present in 67.6% (85/126) of WADII. At 6 months, participants with WADII showed persistent hypoaesthesia to warm, thermal sensory limen and mechanical detection thresholds, and decreased mechanical pain and pressure pain sensitivity compared with controls (*d* > 0.44).

These functional neurological changes were accompanied by elevated serum neurofilament light chain levels in acute WADII compared with controls [*d* = −0.52 (95% confidence interval −0.94, −0.10)]. Intraepidermal nerve fibre densities at the index finger were not significantly different between groups. However, dermal myelin basic protein^+^/protein gene product^+^ myelinated nerve bundles at the index finger were reduced 6 months post-injury in WADII compared with controls [*d* = 0.69 (0.26, 1.11)]. Multivariable linear regression suggested that bedside tests for hypoaesthesia at the index finger were prognostic for whiplash-related upper quadrant pain 6 months post-injury (*r*^2^ = 0.13, *P* = 0.02).

In conclusion, two-thirds of participants with acute WADII initially exhibited signs of neuropathic pain and nerve pathology. At the 6-month follow-up, neuropathic pain persisted in one-third of participants with WADII, and nerve pathology persisted in two-thirds. These findings challenge the traditional musculoskeletal classification of WADII and underscore the need for targeted neurological assessments and treatment.

## Teaser

Fundaun *et al.* investigate grade II whiplash injuries (WADII) and find that two-thirds of patients have signs of acute nerve injury and nerve-related pain, with one-third still affected after 6 months. This challenges the view of WADII as a purely musculoskeletal condition and highlights the need for nerve-focused assessments and treatments.

## Introduction

Non-fatal road traffic collisions impact between 20 and 50 million individuals globally per year.^1^ Whiplash-associated disorders (WADs) commonly result from motor vehicle collisions and pose significant personal and societal challenges.^2^ WAD is a spectrum of symptoms, including pain, psychological distress and sensory/motor dysfunction.^3^ The severity of WAD is commonly graded from zero (no pain and physical signs of injury) to four (neck fracture/dislocation).^4^ The most common type is WAD grade II (WADII), characterized by neck symptoms and musculoskeletal signs (e.g. tenderness and impaired neck movement) in the absence of a frank nerve injury on routine diagnostic testing (traditional neurological examination).^4^ Unfortunately, up to 50% of people with WADII develop persistent pain. Although some prognostic factors for poor recovery have been identified (e.g. increased initial neck-related disability, age and post-traumatic hyperarousal),^5,6^ there is no clear identification of the nature of a structural pathology or pain mechanisms that might underlie chronicity in WADII. The unknown pathology contributing to pain persistence in WADII poses a significant obstacle for developing effective care and improving outcomes for these patients. Indeed, contemporary treatments have small effects at best.^7,8^

Although some studies in WADII demonstrate somatosensory hyperalgesia^9^ and inflammation,^10,11^ signs of nerve pathology (here defined as structural or functional axonal changes leading to loss-of-function) appear to be a common phenotype.^12^ In smaller, chronic WADII cohorts, nerve pathology has been determined structurally using skin biopsies,^13^ through MRI findings suggestive of neuroinflammation,^10^ and functionally using both neurological assessment and quantitative sensory testing (QST).^12^ Despite increasing evidence in the most common type of chronic whiplash injury (WADII), it remains unclear whether such nerve pathology is present acutely after injury and whether it contributes to the development of persistent pain. Identifying nerve pathology and its temporal development in these patients would enhance our understanding of WADII pathomechanisms and help to guide treatment.

The overall aims of this study were to identify the presence (Objective 1) and temporal development (Objective 2) of nerve pathology and to assess their role in prognosis in WADII (Objective 3). We performed comprehensive assessments in people with WADII within 4 weeks of injury, including symptom profiling, clinical neurological assessments, QST, serological and histological skin analyses. Our findings demonstrate that a subgroup of participants with WADII present with signs of neuropathic pain, sensory hypoaesthesia and elevated serological markers of acute axonal injury. Of note, bedside tests for acute cutaneous hypoaesthesia were prognostic for persistent whiplash-related upper quadrant pain but not for neck-related disability 6 months post-injury. Our findings challenge the assumption that WADII solely affects musculoskeletal structures and suggests that a spectrum of nerve pathology might be present and contribute to the pathophysiology for a subgroup of participants with WADII.

## Materials and methods

### Prospective cohort overview

This study is part of a multicentre prospective longitudinal cohort study investigating prognostic factors in WADII. Although in the broader cohort we examined signs of inflammation and nerve mechanosensitivity, this exploratory study focuses specifically on signs of nerve pathology and axonal injury. The study protocol has been published^14^ and was pre-registered at ClinicalTrials.gov (protocol version V2, 25 September 2020; https://clinicaltrials.gov/study/NCT04940923?condWhiplash&rank=10). Testing was completed at university research facilities in Oxford and Brighton, UK. Ethical approval was received from the South Central—Oxford C Ethics Committee (18/SC/0263) and London-Brighton & Sussex Research Ethics Committee (20/PR/0625). All participants gave written informed consent prior to participating. Study data were entered and managed using a REDCap electronic database.^15^ All reporting followed the Strengthening of Reporting of Observational Studies in Epidemiology (STROBE) guidelines for observational cohort studies.^16^

Participants aged 18–85 years in motor vehicle collisions and meeting the Quebec Task Force WADII classification (reduced cervical motion and neck pain without frank neurological signs)^4^ were recruited from local emergency departments. Emergency department staff identified individuals attending after a motor vehicle collision (in person and database search). Those who consented to be contacted for the study were then screened by the research team for eligibility on the telephone and during the baseline appointment. Frank neurological signs were considered if loss of nerve function was present in two or more tests within the same innervation territory upon bedside neurological assessment during the baseline assessment (e.g. loss of both strength and reflexes in C6). Exclusion criteria for participants with WADII included pregnancy; history of cervical/arm pain lasting >3 months; pain from a previous whiplash injury within the previous 12 months; diagnosis of a peripheral neuropathy; and a history of systemic illness known to cause small fibre pathology or neuropathy (e.g. diabetic neuropathy).

Healthy, proportionally age- and sex-matched control participants were recruited to provide normative data for measures of nerve pathology [QST, serum neurofilament light chain (NfL) and histological skin analyses]. Exclusion criteria for healthy control participants included the above criteria in addition to no history of whiplash injury at any time or treatment for cervical/thoracic spine or upper-limb pain within the past 3 months. Additional normative healthy control data for QST, skin biopsies and serum samples were drawn from a previously published cohort^17^ (see Supplementary material).

Baseline assessments for participants with WADII were performed within 4 weeks of whiplash injury and included questionnaires, detailed demographic and medical history, clinical assessment, QST, skin and blood samples. All participants with WADII were invited for follow-up testing 6 months after the date of their whiplash injury, when all baseline measures were repeated plus a validated question regarding their perception of recovery.^18^ Healthy control assessment included a single baseline assessment without questionnaires.

### Clinical phenotyping

#### Self-reported questionnaires

Our selected questionnaires included categories of the six core domains recommended for tracking outcomes following whiplash injury.^19^ Neck-related disability was evaluated using the Neck Disability Index (NDI; 0 is no disability, and 50 is complete disability).^20^ NDI classification of mild <5, moderate 5–14 and severe ≥15 were used, as previously described.^21^

Participants completed a body diagram marking the location of their upper quadrant pain (including the neck and/or upper extremities but excluding headache) resulting from the motor vehicle collision. Participants were instructed to mark their upper quadrant pain corresponding to their whiplash injury within the numbered areas on Supplementary Fig. 1. Participants were then asked to rate the intensity of their marked upper quadrant pain (excluding headache) using a visual analog scale (whiplash-related upper quadrant pain VAS, 0–100 mm). Pain distribution categories (neck versus upper extremity pain) were determined based on body diagram responses: ‘neck pain’ was defined as pain in area 1, and ‘upper extremity pain’ as any pain in areas 2–12. An area was considered painful if any region within it was marked. The number and percentage of participants in each category were calculated for both acute and follow-up time points.

Quality of life was evaluated using the EQ-5D-5L questionnaire,^22^ using the most recently published value set for England,^23^ in the R package eq5d.^24^ Perceived recovery was evaluated using a single question asking, ‘How do you feel you are recovering from your injury?’. This question included six possible responses, spanning from ‘getting much worse’ to ‘all better’, and was shown to be associated with pain, functional limitations and depression in a large cohort of participants post-whiplash injury.^18^

Emotional wellbeing was evaluated using several measures. Trauma-related distress was evaluated using the Impact of Events Scale-Revised (IES-R) and Posttraumatic Stress Disorder-8 (PTSD-8). IES-R scores of ≥33/88 suggest probable PTSD^25^ and PTSD-8 scores of ≥18/32 demonstrated the best predictive value for symptomatic PTSD, which was previously validated in WAD.^26^ Pain-related worrying was assessed using the Pain Catastrophizing Scale (PCS, cut-off score of ≥30/52 for significant pain-related worrying).^27^ Negative emotional states were measured using the Depression, Anxiety and Stress Scale (DASS42). The three DASS42 subscales are scored out of 42, with mild scores <6, moderate 7–12 and severe ≥13.^28^

#### Neuropathic pain grading

The certainty of neuropathic pain was classified for each WADII participant according to the updated Neuropathic Pain Grading System published by the Neuropathic Pain Special Interest Group (NeuPSIG) of the International Association for the Study of Pain.^29^ In brief, the grading of possible neuropathic pain included a history of whiplash injury, with pain descriptors and symptom behaviour related to neuropathic pain (e.g. burning, tingling, electric shocks, cold-like pain and spontaneous pain) and a neuroanatomically plausible pain distribution, including over the neck or upper extremities. Probable neuropathic pain included negative sensory signs or allodynia in the main pain area identified through bedside neurological assessment. A grading of probable neuropathic pain could be reached only if the previous categories were met. We decided against the inclusion of the category of definite neuropathic pain because MRI and index finger skin biopsies were not available in every participant, and the latter also lack age-corrected normative values. Furthermore, both probable and definite neuropathic pain would follow the same management strategies according to the neuropathic pain guidelines.^29^ Participants were characterized as ‘unlikely’ if no neuropathic pain descriptors were reported and/or if the pain distribution was not neuroanatomically plausible.

#### Bedside neurological assessment

Neurological assessment of the bilateral upper extremities was performed within the C5–T1 innervation territories, including reflexes, myotomal strength, cutaneous light touch (cotton wool), pinprick (neurotip) and thermal sensation (coins) (Supplementary Table 1). A composite classification of neurological assessment results (accounting for all innervation territories) was created for individual participants, labelled as normal (if no loss- or gain-of-function was present), loss-of-function (any measures of reduced sensation, strength or reflexes), gain-of-function (any measures of increased response to sensation or reflex testing) or mixed (if participants showed signs of both loss- and gain-of-function).

#### Quantitative sensory testing

The standardized QST protocol was performed according to the German Network for Neuropathic Pain^30^ on the ventral aspect of the proximal phalanx of the index finger. This location has previously shown changes in chronic WADII.^13,31,32^

Warm and cold detection thresholds, cold and heat pain thresholds, and thermal sensory limen were measured using a Thermotester (Somedic) with a 25 mm × 50 mm thermode. Mechanical and vibration detection thresholds were measured using von Frey filaments (Optihair 2 MRC Systems) and a Rydel Seiffer tuning fork (over the second metacarpophalangeal joint), respectively. Pressure pain thresholds were assessed using a hand-held algometer over the thenar eminence (Wagner Force dial) and mechanical pain sensitivity using weighted pinprick stimulators (MRC Systems). The number of paradoxical heat sensations during thermal sensory limen and the presence of dynamic mechanical allodynia were quantified.

Participants with WADII were tested on the most symptomatic side; healthy controls were tested on their non-dominant side. Additionally, cold and warm detection and pressure pain thresholds were performed over the contralateral lower limb (upper anterolateral aspect of the tibia) to assess for potential widespread sensory changes. The QST protocol has shown high intra-tester and good inter-tester reliability,^33-35^ with good long-term stability.^35^ QST data were *z*-transformed for analysis (see Supplementary material).

#### Histological skin analysis

Two 3-mm-diameter skin biopsies were obtained aseptically from the ventrolateral aspect of the proximal phalanx of the index finger under local anaesthesia (1% lidocaine, 1–2.0 ml). We selected the index finger on the most symptomatic side for participants with WADII, because it has previously demonstrated small fibre pathology in chronic WADII.^13^ Biopsies from healthy controls were taken from the non-dominant index finger. We also took skin biopsies 10 cm above the contralateral lateral malleolus in both groups. Biopsies were fixed in periodate–lysine–paraformaldehyde for 30 min, followed by washing in 0.1 M PBS, and incubated in 15% sucrose at 4°C for 3 days. The tissue was embedded in OCT compound and stored at −80°C.

Fifty-micrometre-thick sections were cut and immunohistochemistry was performed, as previously described.^36^ In brief, free-floating skin sections were blocked in 5% fish gelatine for 1 h. Next, primary antibodies for protein gene product 9.5 (PGP9.5, Zytomed 1:200) and myelin basic protein (MBP, Abcam 1:500) were incubated overnight. After three 1 h wash cycles in PBS, secondary antibodies were incubated overnight [Alexa 488, 1:1000, Abcam (catalogue no. ab150157); Cy3, 1:500, Stratech (Cat. no. 712-165-153-JIR)]. The next day, sections were mounted, counterstained for nuclei with 4′,6-diamidino-2-phenylindole and coverslipped.

Intraepidermal and dermal nerve fibre integrity were evaluated down a confocal microscope (Zeiss Axio LSM 700) following established protocols^17,36,37^ by an investigator blinded to the condition of the participant and time point. Intraepidermal nerve fibre densities, averaged from three samples per participant, were expressed as epidermal fibres per millimetre. Dermal fibre integrity was determined by the number of dermal nerve bundles per millimetre squared containing MBP and at least five PGP9.5^+^ fibres, excluding the subepidermal plexus.^17,36^ Meissner corpuscles were counted and expressed per millimetre of epidermis.^36^ All measures were calculated for both finger and leg biopsies, except Meissner corpuscles, which are present only in glabrous skin (e.g. finger).^38^

#### Serum marker of axonal injury

Blood was collected from the cubital fossa (BD Vacutainer Tube SST Advance) and centrifuged at 1300 g at 4°C for 10 min. The serum fraction was collected and stored at −80°C for batch analysis. Protein concentrations of NfL were analysed using the Simoa SR-X and NF-Light v.2 Advantage Kit (Quanterix). All samples were analysed in duplicate with a randomized combination of healthy controls and paired acute and follow-up WADII samples on each plate. A calibration curve was included on each plate.

Serum NfL levels were initially calculated from the mean absolute concentration (in pg/ml) averaged from duplicate samples. Absolute concentrations were then transformed into *z*-scores for age and body mass index, as previously described.^39^ Normative data for *z*-score transformation were derived from a large general population cohort without diagnosis of CNS disease or other major diseases (*n* = 4532 control participants).^39^ To adjust for the slight divergence in kits used between our study and the general population cohort (NF-light assay v.2 versus v.1), Nf-light v.2 concentrations were multiplied by a factor of 0.89, according to technical recommendations from the supplier (Simoa, Quanterix).

### Statistical analysis

Sample size calculations revealed that we needed a minimum of *n* = 69 participants with WADII and *n* = 42 healthy control participants to be sufficiently powered for our objectives. For detailed estimations, please refer to the Supplementary material.

Data were analysed using R software (v.4.0.3). Data normality was assessed using the Kolmogorov–Smirnov test and visual inspection. Clinical phenotypic data were presented as the mean and standard deviation (SD) for normally distributed data and as the median and interquartile range (IQR) for non-normally distributed data. The fraction of total missing data was <5%, hence missing values were discarded, and imputation procedures were not required.^40,41^ Statistical significance was set at *P <* 0.05.

Neurological assessment results were reported descriptively and presented as the percentage of individuals with normal function, loss-of-function or gain-of-function. To evaluate the overlap of acute variables of nerve pathology and neuropathic pain, cut-off thresholds were established to create a Venn diagram integrating results for acute participants with WADII with a complete dataset for all included parameters. Venn diagram parameters and corresponding cut-off thresholds included: NfL *z*-score >1.5^39^; QST *z*-scores of < −1.96 in at least one parameter^42^; at least possible neuropathic pain using the NeuPSIG grading system^29^; and bedside neurological assessment including at least one measure with loss-of-function.

Temporal measures of nerve pathology (QST, intraepidermal nerve fibre densities and NfL) were compared among proportionally age- and sex-matched healthy controls, acute and follow-up participants with WADII using one-way ANOVA with Tukey's *post hoc* testing for parametric comparisons or aligned ranks transformation ANOVA with Tukey's *post hoc* testing for non-parametric comparisons. We also report effect sizes (e.g. Cohen's *d*) and 95% confidence intervals.

We examined the prognostic role of nerve pathology in WADII using multivariable linear regression. The NDI served as the clinical end point, consistent with its extensive use in WAD prognosis,^43-45^ and was treated as a continuous variable. We examined the preplanned prognostic role of intraepidermal nerve fibre densities and performed secondary exploratory analyses of serum NfL levels and bedside sensory measures of nerve pathology separately. NfL age- and body mass index-adjusted *z*-scores were chosen as an additional structural measure of nerve pathology. Light touch, thermal and pinprick cutaneous loss-of-function at the index finger served as surrogate bedside measures of functional nerve pathology.^46^ The cumulative value of bedside sensory loss-of-function, obtained by summing the number of hypoalgesic responses to light touch (cotton wool), thermal and pinprick stimuli across bilateral index fingers for each participant, was analysed using multivariable linear regression. Previous research has indicated age, sex and initial NDI scores can influence recovery in WAD.^5,47^ Consequently, these were included as covariates in the linear regression models. In addition to the NDI primary end point, we also examined each multivariable linear regression model using whiplash-related upper quadrant pain at follow-up (VAS) as a secondary clinical end point. Additional information is provided in Supplementary material.

## Results

Our cohort consisted of 129 acute participants with WADII [median age 36.0 years (IQR = 22.0), 58% (75/129) female] and 36 healthy controls [median age 39.0 years (IQR = 25), 61% (22/36) female; Table 1]. Of the 97 participants with WADII who completed follow-up questionnaires, 67 also attended an in-person follow-up assessment (Supplementary Fig. 2). Moderate NDI was present acutely (median = 15.0, IQR = 10.0) and was significantly reduced at 6 months (median = 7.0, IQR = 10.3, *P* < 0.0001, *W* = 9409.5). Median whiplash-related upper quadrant pain was rated as 35.0/100 acutely (IQR = 33.3) and 17.0/100 (IQR = 29.5, *P* < 0.001, *W =* 6242.5) at 6 months (Table 1). Complete details are provided in Supplementary Table 2.

**Table 1:** Demographic and clinical characteristics of the study population

Characteristic	Healthy controls^a^	Acute WADII	Follow-up WADII	*P*-value (acute versus follow-up)
Count	36	129	97 completed questionnaires 67 attended in-person assessment	–
Age (median/IQR), years	39.0 (25.0)	36.0 (22.0)	–	–
Female sex (median/IQR)	61% (22/36)	58% (75/129)	–	–
BMI (median/IQR), kg/m^2^	24.3 (5.1)	26.5 (6.5)	–	–
Neck Disability Index (median/IQR; total 50)	–	15.0 (10.0)	7.0 (10.3)	<0.001
Mild	–	4% (5/126)	32% (31/96)	–
Moderate	–	42% (53/126)	47% (45/96)	–
Severe	–	54% (68/126)	21% (20/96)	–
Current whiplash-related upper quadrant pain VAS (median/IQR)	–	35.0 (33.3)	17.0 (29.5)	<0.001
Mild (<30/100)	–	39% (50/128)	66% (43/65)	–
Moderate–severe (≥30/100)	–	61% (78/128)	34% (22/65)	–
Average pain (median/IQR)	–	6.0 (2.0) *n* = 119	3.0 (3.0) *n* = 94	<0.001
Current pain (median/IQR)	–	4.0 (3.5) *n* = 119	2.0 (3.0) *n* = 94	<0.001
Strongest pain (median/IQR)	–	8.0 (2.0) *n* = 119	4.0 (5.0) *n* = 94	<0.001
PainDETECT median/IQR)	–	9.0 (9.0)	6.0 (8.0)	<0.001
NeuPSIG Grading				
Unlikely	–	37.5% (48/128)	68.2% (45/66)	–
Possible	–	27.3% (35/128)	10.6% (7/66)	–
Probable	–	35.2% (45/128)	21.2% (14/66)	–

Numerical data are presented as the median (IQR) when not normally distributed, the mean (SD) when normally distributed, or a percentage (%) for categorical data. Neck Disability Index scoring: mild <5; moderate 5–14; and severe ≥15. Average, current and strongest pain levels over past 4 weeks were taken from the corresponding questions within the painDETECT questionnaire. Healthy control participants did not complete self-reported outcome measures and, by definition of inclusion criteria, did not have any upper quadrant symptoms. Wilcoxon signed-rank tests were used for all non-parametric comparisons, and a *t*-test was used for normally distributed variables. Bold text indicates statistical significance (set at *P <* 0.05). The additional healthy control participants for quantitative sensory testing analysis (*n* = 67 total) are described in the Supplementary material. BMI = body mass index; IQR = interquartile range; NeuPSIG = Neuropathic Pain Special Interest Group; SD = standard deviation; VAS = visual analog scale; WADII = whiplash-associated disorder grade II.

^a^Indicates healthy controls who were age matched for neurofilament light chain analysis and age and sex matched for histological skin analyses.

### Acute neuropathic pain is present in up to two-thirds of WADII and persists 6-months post-injury

**Figure 1:**
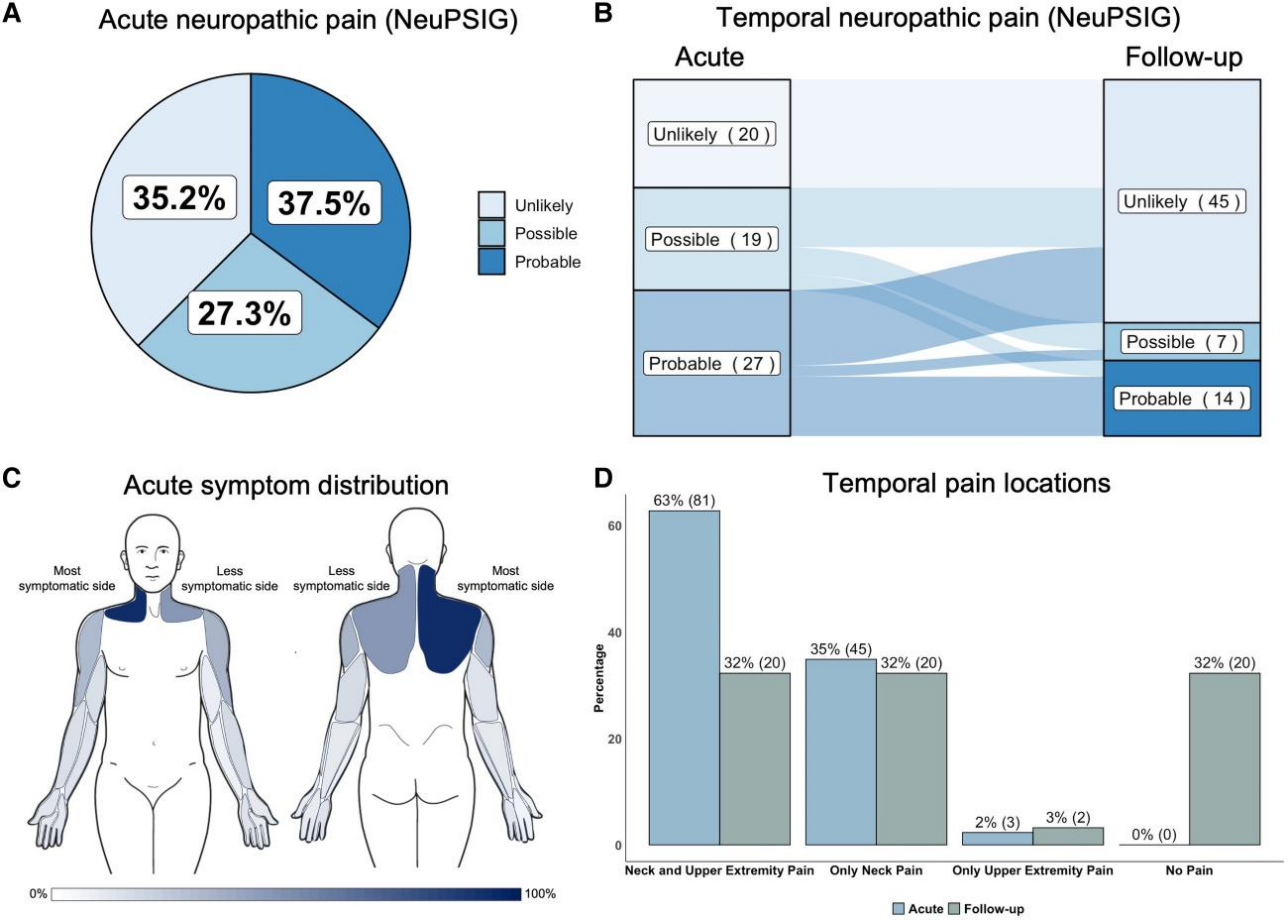
**Neuropathic pain is present acutely and 6 months after whiplash injury in a subgroup of participants with WADII.** (**A**) Distribution of the certainty of neuropathic pain for acute participants with WADII using the NeuPSIG grading system (*n* = 129). (**B**) Temporal patterns of the certainty of neuropathic pain in participants with WADII with paired acute and follow-up findings (*n =* 66) using the NeuPSIG grading system. (**C**) The body diagram shows the percentage and location of whiplash-related upper quadrant pain for acute participants with WADII. (**D**) Percentage of upper quadrant pain distribution for acute and follow-up participants with WADII, with data presented as a percentage (number of participants). NeuPSIG = Neuropathic Pain Special Interest Group; WADII = whiplash-associated disorder grade II.

Sixty-five per cent of acute participants with WADII presented with at least possible neuropathic pain features (Fig. 1A). Six-month follow-up assessment revealed signs of persistent neuropathic pain in 31.8% (21/66) of participants with WADII (Fig. 1B). Acute pain was most commonly located in both the neck and the upper extremities (63%, 81/129), while 35% (45/129) reported pain limited to the neck (Fig. 1C and D). At the 6-month follow-up, 32% (21/67) reported persistent pain in both the neck and upper extremities, with another 32% (21/67) reporting pain confined to the neck. Subjective reports of sensory changes, such as numbness or tingling, were present in 42% (54/128) of acute WADII participants and persisted in 30% (17/57) at 6 months (Supplementary Fig. 3).

### Acute nerve pathology upon bedside neurological assessment is common in WADII

**Figure 2:**
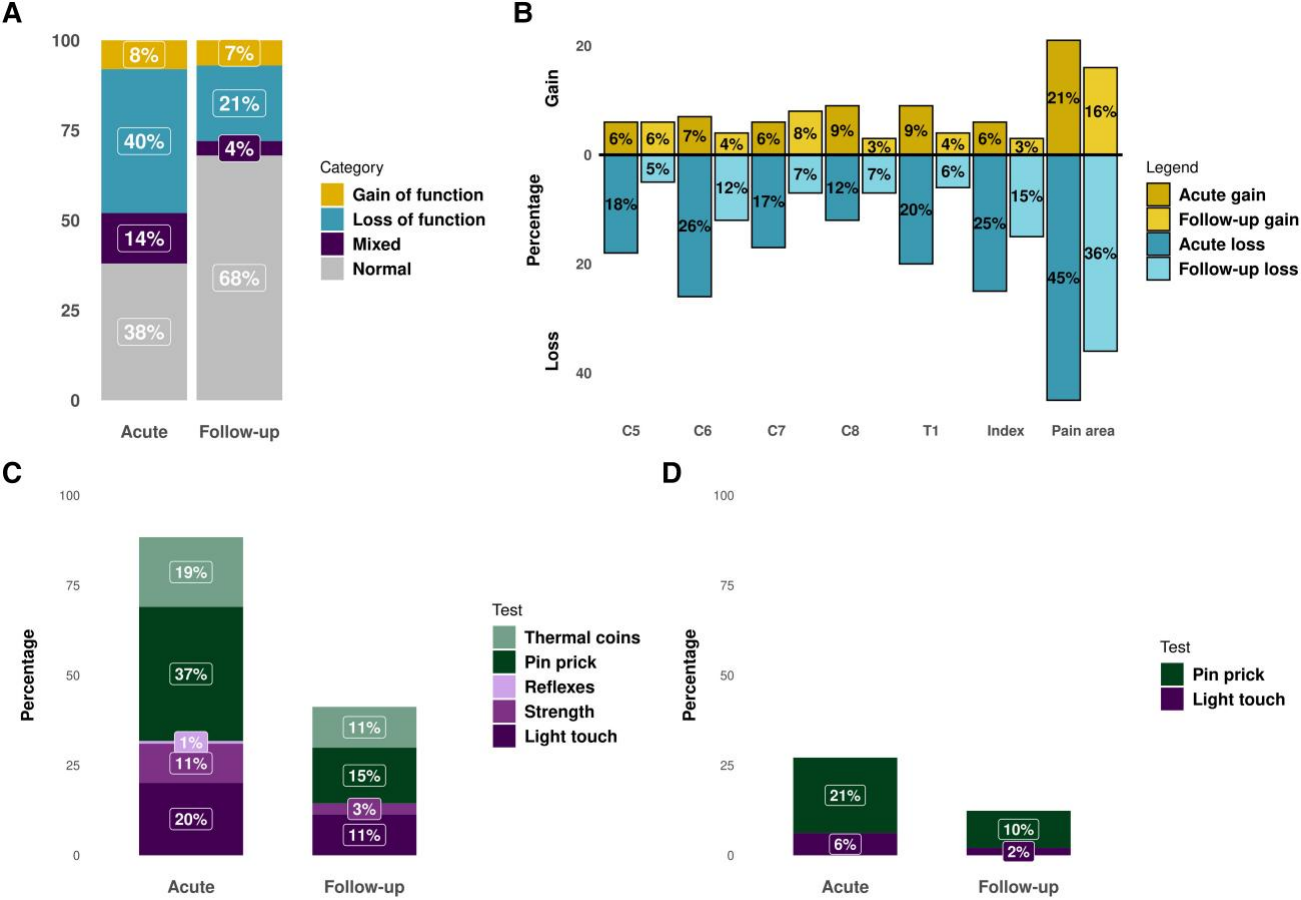
**Bedside neurological assessment identifies loss-of-function in 54% of participants with whiplash-associated disorder grade II in the acute stage persisting throughout the upper extremity in some individuals at 6 months**. (**A**) Categorization of acute and follow-up composite neurological assessment, including strength, reflexes, light touch, pinprick and thermal coins. (**B**) Percentage of composite neurological dysfunction in C5–T1 innervation territories of the most symptomatic side, index finger and main pain area for acute and follow-up, including strength, reflexes, light touch, pinprick and thermal coins. (**C** and **D**) Type and percentage of bedside neurological tests contributing to measures of loss-of-function (**C**) and gain-of-function (**D**) at both time points. Purple colours indicate large nerve fibres, and green colours indicate small nerve fibres. *n* = 129 acute and *n* = 67 follow-up participants.

Bedside neurological assessment revealed predominantly neurological loss-of-function (Fig. 2A). The primary painful area on the most symptomatic side of participants with WADII had the highest percentage of participants with acute loss-of-function (45%), which decreased to 36% of participants at follow-up (Fig. 2B). When considering specific innervation territories, C6 had the highest prevalence of loss-of-function, affecting 26% of acute participants with WADII and 12% at follow-up (Fig. 2B). Nerve pathology upon bedside neurological testing was less prevalent at all levels contralateral to the primary painful side of participants (Supplementary Fig. 2). Loss-of-function was predominantly from small nerve fibres (warm/cold or pinprick), which accounted for 56% of abnormal tests acutely and 26% at follow-up (Fig. 2C). Gain-of-function was also mainly from small nerve fibres (pinprick), accounting for 21% of abnormal tests acutely and 10% at follow-up (Fig. 2D).

### QST reveals sensory hypoaesthesia in two-thirds of participants with WADII persisting at 6 months

**Figure 3:**
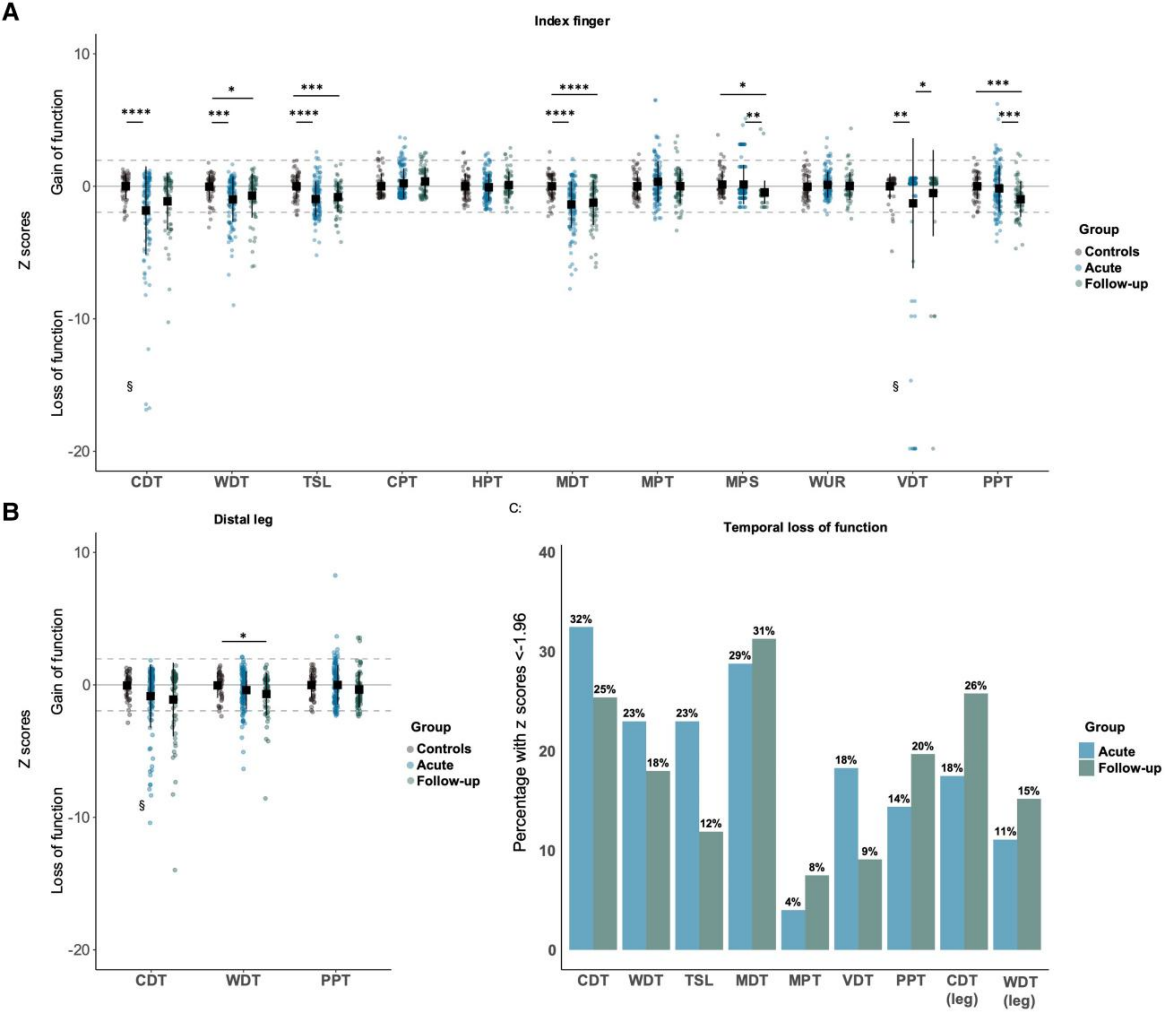
**Acutely reduced sensory detection thresholds over the index finger persist 6 months after injury.** (**A**) QST measured over the index finger in WADII and controls. To improve figure readability, *y*-axis values are limited to −20, resulting in some *z*-scores outside of the range (indicated as §). Unplotted index finger CDT *z*-score for acute WADII = −26.7. The unplotted index finger VDT *z*-scores for acute WADII = −79.9, −59.8, −39.8, −29.8, −29.8, −29.8, −29.8, −29.8, −29.8 and −20.7. Unplotted index finger follow-up VDT *z*-score = −29.8. (**B**) QST measured over the tibialis anterior in WADII and controls. Owing to *y*-axis limitations, three acute WADII CDT *z*-scores at the distal leg are outside the figure range (indicated as §). Unplotted CDT *z*-scores = −40.4, −26.4 and −20.6. Index finger data include *n* = 126 acute WADII, *n* = 67 follow-up WADII and *n* = 61 controls. Leg data include *n* = 113 acute WADII, *n* = 61 follow-up WADII and *n* = 46 controls. (**C**) Temporal loss-of-function expressed as the percentage of patients presenting with *z*-scores < −1.96. PPT is included owing to the presence of hypoaesthesia to PPT over the hand for a subgroup at 6-month follow-up. One-way ANOVA tests with Tukey's honest significant difference *post hoc* testing were used for group comparisons. Black square indicates the mean, with extending lines indicating one standard deviation from the mean. *P < 0.05; **P < 0.01; ***P < 0.001; ****P < 0.0001. CDT = cold detection threshold; CPT = cold pain threshold; HPT = heat pain threshold; MDT = mechanical detection threshold; MPS = mechanical pain sensitivity; MPT = mechanical pain threshold; PPT = pressure pain threshold; QST = quantitative sensory testing; TSL = thermal sensory limen; VDT = vibration detection threshold; WADII = whiplash-associated disorder grade II; WDT = warm detection threshold; WUR = wind-up ratio.

There were reduced cold and warm detection thresholds, thermal sensory limen, mechanical and vibration detection thresholds in acute WADII compared with controls, with small to large effects (*d* = 0.47–0.87; Fig. 3A and Supplementary Tables 3, 4 and 7). Acute loss-of-function in at least one QST parameter (defined as a *z*-score < −1.96) was present in 67.6% (85/126) of participants with WADII. Hypoaesthesia to cold and mechanical detection over the index finger were the most common impairments in acute participants with WADII, affecting 32.5% (41/126) and 29.4% (37/126), respectively (Fig. 3C).

At 6-month follow-up, warm detection thresholds, thermal sensory limen, mechanical detection thresholds, mechanical pain sensitivity and pressure pain thresholds continued to be reduced in participants with WADII compared with controls, with small to medium effects (*d* = 0.44–0.69; Fig. 3A). There were also reduced mechanical and pressure pain thresholds and reduced vibration detection thresholds at follow-up compared to the acute stage (*d* = 0.37–0.55). Warm detection over the leg was reduced in follow-up WADII compared with controls (*d* = 0.47; Fig. 3B). Loss-of-function in at least one QST parameter (defined as a *z*-score < −1.96) increased to 74.6% (50/67) at follow-up. Hypoaesthesia to mechanical and cold detection over the index finger was the most common impairment at follow-up, affecting 31% (20/67) and 25% (17/67) of participants with WADII, respectively (Fig. 3C).

### Intraepidermal nerve fibre densities are preserved but dermal bundles are reduced at 6 months

**Figure 4:**
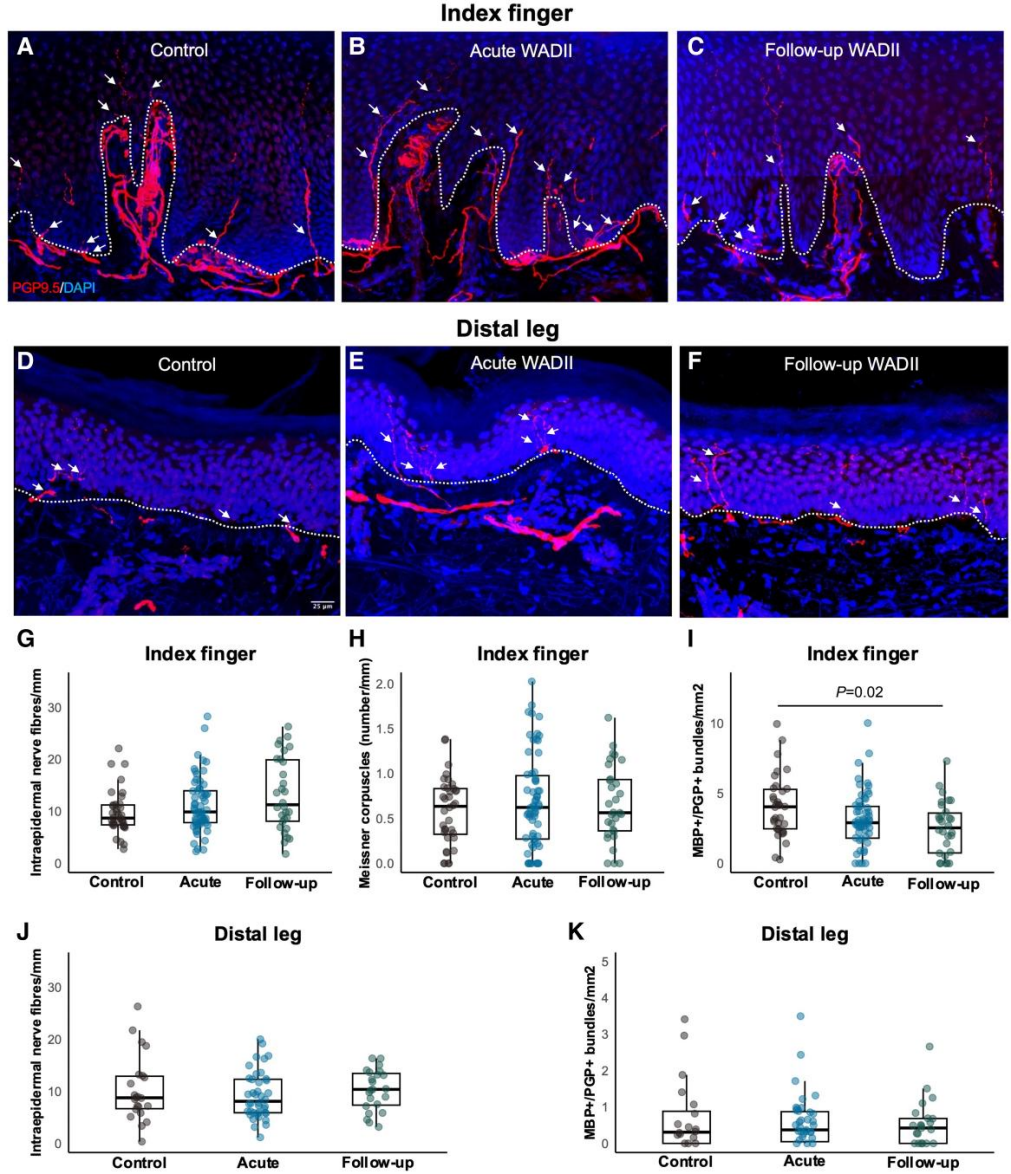
**Cutaneous innervation of the index finger and leg is preserved in the acute stage, with reduced dermal nerve bundles at the index finger at 6 months.** (**A–F**) Representative images of skin immunohistochemistry taken at the index finger and distal leg in a participant with acute WADII and follow-up WADII, and in an age- and sex-matched healthy control. Arrows indicate epidermal small nerve fibres, and the dashed lines indicate the dermal–epidermal border. (**G**) Comparison of intraepidermal nerve fibre densities at the index finger in WADII and controls (median and IQR). (**H**) Comparison of Meissner corpuscles at the index finger in WADII and controls (median and IQR). (**I**) Comparison of MBP^+^ dermal bundles with at least five PGP^+^ nerve fibres at the index finger in WADII and controls (median and IQR). (**J**) Comparison of intraepidermal nerve fibre densities at the distal leg in WADII and controls. (**K**) Comparison of MBP^+^ dermal bundles with at least five PGP^+^ nerve fibres at the leg in WADII and controls (median and IQR). Aligned ranks transformation ANOVA with Tukey's *post hoc* testing was used for all comparisons. Total index finger samples used for analysis include *n* = 62 acute WADII, *n* = 30 follow-up WADII and *n* = 36 controls. Total leg samples used in analysis include *n* = 39 acute WADII, *n* = 25 follow-up WADII and *n* = 22 controls. IQR = interquartile range; MBP = myelin basic protein; PGP = protein gene product; WADII = whiplash-associated disorder grade II.

There were no differences in intraepidermal nerve fibre densities between acute WADII, follow-up WADII and controls at the index finger [*F*(2,12) = 2.29, *P* = 0.11] or the distal leg [*F*(2,82) = 0.64, *P* = 0.53; Fig. 4A–G and J and Supplementary Table 5. Likewise, there were no significant differences in the number of Meissner corpuscles at the index finger between acute WADII, follow-up WADII and controls [*F*(2,82) = 0.19, *P* = 0.82; Fig. 4H].

There was a significant reduction in the number of dermal MBP^+^/PGP^+^ myelinated nerve bundles at the index finger when comparing controls and follow-up participants with WADII [*t*(121) = 2.7, *P* = 0.02, *d* = 0.69 (95% CI: 0.26, 1.11); Fig. 4I], with a non-significant trend in the acute stage [*t*(121) = 2.2, *P* = 0.08, *d* = 0.47 (95% CI: 0.05, 0.89)]. There were no significant differences in MBP^+^/PGP^+^ myelinated nerve bundles at the index finger when comparing acute with follow-up WADII [*t*(121) = 0.95, *P* = 0.61, *d* = 0.21 (95% CI: −0.20, 0.63); Fig. 4I]. There were also no significant differences in MBP^+^/PGP^+^ bundles at the leg between any of the groups [control versus acute WADII, *t*(79) = −0.29, *P* = 0.95, *d* = −0.08 (95% CI: −0.61, 0.45); control versus follow-up WADII, *t*(79) = 0.08, *P* = 0.99, *d* = 0.02 (−0.51, 0.55); and acute versus follow-up WADII, *t*(79) = 0.39, *P* = 0.92, *d* = 0.10 (−0.43, 0.63); Fig. 4K].

### Acutely elevated NfL levels resolve 6 months after injury

**Figure 5:**
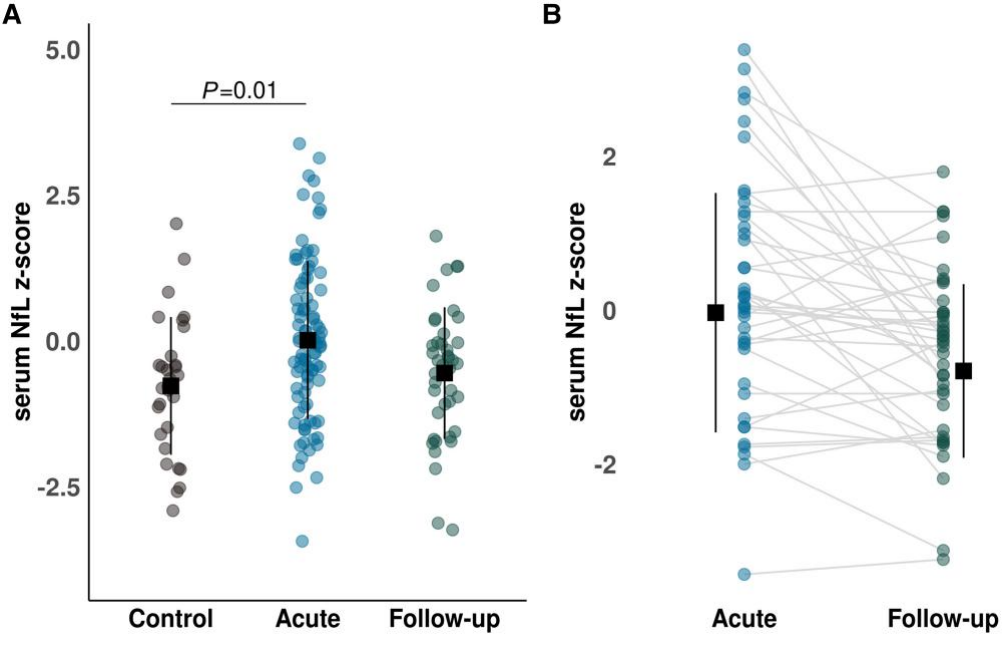
**Serum NfL levels are acutely elevated after whiplash injury.** (**A**) Serum NfL *z*-scores in WADII and proportionally age-matched control participants were compared using a one-way ANOVA with Tukey's honest significant difference *post hoc* testing. *n* = 30 controls, *n* = 91 acute WADII and *n* = 41 follow-up WADII. (**B**) Paired serum NfL *z*-scores for acute and follow-up participants with WADII (*n* = 41). Black square indicates the mean, with extending line indicating one standard deviation from the mean. NfL = neurofilament light chain; WADII = whiplash-associated disorder grade II.

NfL *z*-scores were significantly elevated in acute WADII compared with controls [mean difference = −0.78, 95% CI (−1.41, −0.15), *P* = 0.01, *d* = 0.62 (95% CI: 0.20, 1.04); Fig. 5A and Supplementary Table 6. There were no significant differences between follow-up WADII and controls [mean difference = 0.22, 95% CI (−0.50, 0.94), *P* = 0.75, *d* = 0.17 (95% CI: −0.65, 0.30)] or acute and follow-up participants with WADII [mean difference = −0.56, 95% CI (−1.13, 0.00), *P* = 0.05, *d* = 0.44 (95% CI: 0.07, 0.82); Fig. 5A and B].

### Acute WADII exhibits substantial overlap in neuropathic pain and nerve pathology

**Figure 6:**
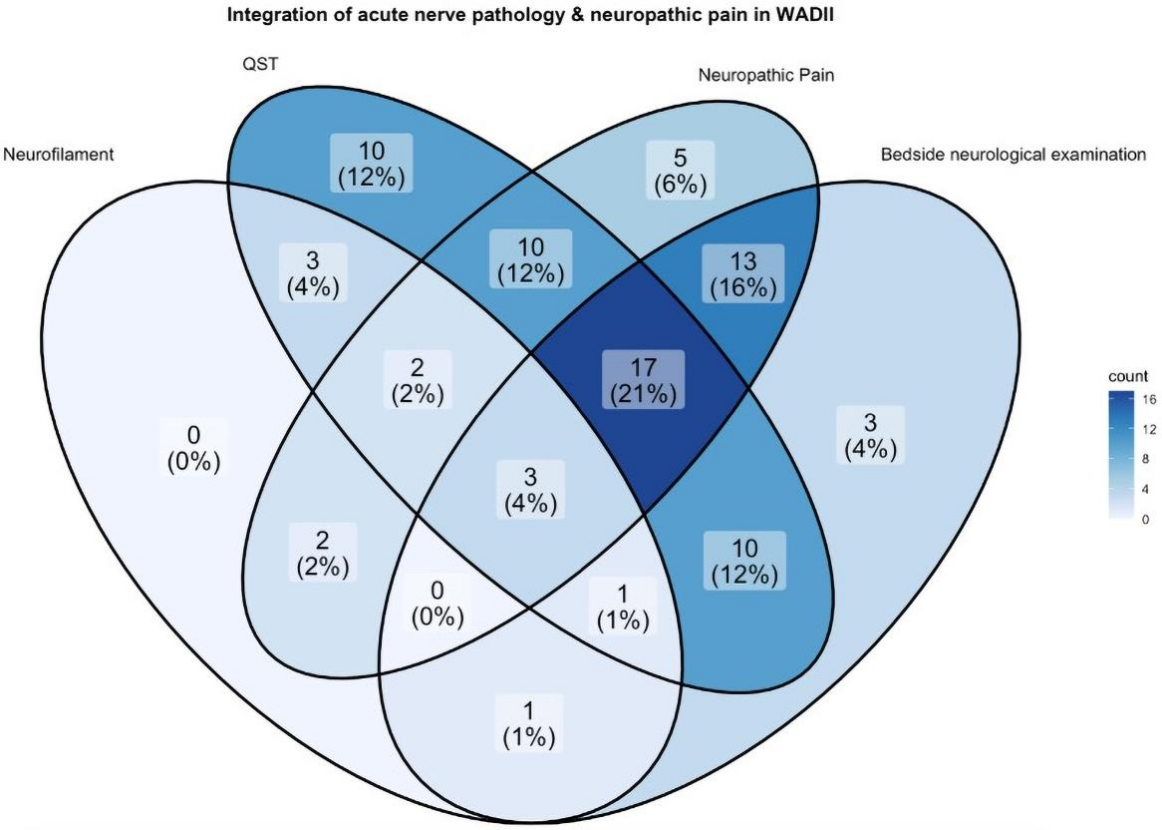
**Aggregation of participants with WADII with signs of acute neuropathic pain and nerve pathology.** Cut-off thresholds indicative of nerve pathology, as previously described, included: neurofilament light chain *z*-score > 1.5; QST *z*-scores of < −1.96 in at least one parameter; at least possible neuropathic pain using the NeuPSIG grading system; and bedside neurological assessment including at least one measure with loss-of-function. The analysis included *n* = 88 participants with complete datasets, of whom *n* = 80 had measures indicative of neuropathic pain and nerve pathology based on these thresholds. NeuPSIG = Neuropathic Pain Special Interest Group; QST = quantitative sensory testing; WADII = whiplash-associated disorder grade II.

Analysis using a Venn diagram revealed that the most common combination of acute findings was signs of neuropathic pain based on the NeuPSIG grading paired with abnormal QST and bedside neurological assessments in 21% (17/80) of participants with WADII (Fig. 6). Among participants with WADII with acute neuropathic pain based on the NeuPSIG grading, only 6% (5/80) exhibited signs of neuropathic pain without any additional evidence of nerve pathology. Notably, all acute participants with WADII with abnormally high NfL *z*-score values also showed evidence of neuropathic pain or nerve pathology.

### Sensory hypoaesthesia from bedside measures is prognostic for whiplash-related upper quadrant pain at 6 months

Multivariable linear regression analysis confirmed that the *a priori* selected acute measures of nerve pathology in WADII were not prognostic for neck-related disability at 6 months (Table 2). Secondary analyses revealed that bedside tests for loss-of-function at the index finger [but not intraepidermal nerve fibre densities (*P* = 0.80) or acute serum NfL *z*-scores (*P* = 0.81)] were significant in explaining 13% of the observed variance of whiplash-related upper quadrant pain (*P* = 0.02).

**Table 2:** Clinical loss-of-function predicts whiplash-related upper quadrant pain but not neck-related disability at 6 months using multivariable linear regression

Variable	Number of participants in model	Coefficient estimate	Standard error	*P*-value	Overall model adjusted *R*^2^ value
Neck-related disability					
IENFD	52	−0.10	0.16	0.55	0.33
NfL *z*-scores	69	0.15	0.47	0.74	0.28
Clinical loss-of-function (index finger)	94	0.01	2.24	0.99	0.39
Whiplash-related upper quadrant pain					
IENFD	36	−0.28	0.72	0.70	0.03
NfL *z*-scores	48	0.45	1.84	0.81	0.12
**Clinical loss-of-function (index finger)**	**64**	**7.16**	**2.88**	**0.02**	**0.13**

Each multiple linear regression model included follow-up WADII Neck Disability Index scores or whiplash-related upper quadrant pain measured by VAS as the dependent variable, with NfL or clinical loss-of-function as the independent variables, controlling for age, sex and initial neck-related disability. Bold text indicates statistical significance. IENFD = intraepidermal nerve fibre density; NfL = neurofilament light chain; WADII = whiplash-associated disorder grade II.

## Discussion

The primary aims of this study were to examine the presence and temporal development of neuropathic pain and nerve pathology and to assess their prognostic role in WADII. Our findings challenge the long-held view that WADII is purely musculoskeletal, revealing a spectrum of neurological involvement and diverse clinical phenotypes. We demonstrate, for the first time, acute structural axonal injury in a subset of participants with WADII, evidenced by elevated NfL levels. Acute neuropathic pain and functional nerve pathology were also identified in approximately two-thirds of participants, with sensory hypoaesthesia quantified by QST over the index finger persisting in nearly 75% at 6 months. Acute sensory hypoaesthesia measured with low-cost bedside tests was prognostic of persistence of whiplash-related upper quadrant pain. This prognostic significance emphasizes the importance of evaluating these measures in people with WADII acutely after injury. Our findings suggest that nerve pathology in the acute phase after whiplash injury is not merely a consequence of chronic symptoms but can be an immediate result of the injury for some participants, suggesting a reconsideration of the clinical approach to and classification of whiplash injuries.

### Acute neuropathic pain is a common feature in participants with WADII

The presence of at least possible neuropathic pain using the NeuPSIG grading was evident in two-thirds of acute participants with WADII, suggestive of nerve involvement in the acute stages following a whiplash injury. Our previous systematic review, which graded neuropathic pain at study level, demonstrated similarly high rates in mostly chronic WADII.^12^ In this study, neuropathic pain in the acute stage was accompanied by clear evidence of nerve pathology in the majority of participants, as indicated by abnormalities in neurological assessments and QST and by elevated NfL levels. The presence of acute neuropathic pain in WADII has important clinical implications, because it often requires different treatment strategies from non-neuropathic conditions.^29^

### Acute structural and functional nerve pathology suggest potential preganglionic injury

Our study provides evidence for an acute structural nerve pathology in a subset of participants with WADII using serological NfL levels, indicative of acute axonal injury, challenging the notion of WADII injuries as solely musculoskeletal. Given that NfL can be upregulated by axonal damage in both the central and peripheral nervous system,^48^ these findings alone cannot pinpoint the exact location of axonal injury. The aggregation of nerve pathology signs in our cohort, however, cannot exclude a potential acute preganglionic injury [e.g. (multiple) cervical dorsal nerve roots, spinal cord] in a subgroup of participants with WADII. Key findings supporting this hypothesis include acute upper extremity hypoaesthesia in two or more innervation territories in >50% of participants with WADII, while intraepidermal nerve fibre densities at the index finger remained preserved.

The cervical dorsal roots might be particularly vulnerable to acute injury owing to their anatomical characteristics and susceptibility to rapid spinal movement.^49-51^ Furthermore, subtle signs of spinal cord pathology have previously been reported in chronic, non-recovered participants with WADII, including changes in spinal cord white matter tracts observed using MRI and impaired voluntary plantar flexor activation.^52-54^ However, based on our exclusion criteria, it is unlikely that our participants had a frank spinal cord injury. Although these findings suggest that a structural preganglionic component might be present in a subset of participants with WADII, postganglionic involvement might also be possible. Albeit not significant, our skin biopsy analysis showed a trend towards acutely reduced dermal innervation. This was probably an underpowered secondary analysis, requiring further investigation. As such, WADII injuries might involve a complex interplay of pre- and postganglionic elements, necessitating a more nuanced approach to classification and treatment.

Our findings of acute functional nerve pathology, based on neurological assessments and QST, corroborate these structural changes. More than two-thirds of participants with WADII presented with signs of acute somatosensory hypoaesthesia in the upper extremity. Although previous WAD studies have mainly examined acute somatosensory gain-of-function (e.g. hyperalgesia over the cervical spine^9^), evidence for acute loss-of-function is limited, despite it being a hallmark of nerve pathology.^29^ Our findings align with acute hypoaesthesia to cold, vibration and electric detection thresholds identified within the C6–C7 innervation territories after acute WADII.^31^ In addition to multisegmental changes upon neurological examination, we also noted a trend for reduced warm detection thresholds over the distal leg, which was significant in the chronic stage despite preserved intraepidermal nerve fibre density.

### Neuropathic pain improves but does not resolve 6 months after injury

At 6 months, persistent neuropathic pain was present in one-third of participants with WADII. Notably, persistent neuropathic pain was present only in participants with WADII who presented with acute neuropathic pain and did not develop *de novo*. Although no previous WAD cohorts have tracked neuropathic pain longitudinally, our findings align with a study of participants after traumatic musculoskeletal injury, which suggested that 21% of participants with acute neuropathic pain continued to have moderate to severe neuropathic pain at 4-month follow-up.^55^ The persistence of neuropathic pain into the chronic stages underscores the importance of the early identification and temporal monitoring in WADII. Despite overall signs of improvement, the presence of persistent neuropathic pain in WADII suggests that a subgroup might require more targeted neuropathic management strategies.

Previous research highlights clinical features indicative of central sensitization and characteristics of nociplastic pain, including widespread pain and hypersensitivity in chronic, non-recovered WAD.^56^ However, our 6-month follow-up using pressure pain thresholds over the leg did not indicate widespread hypersensitivity at the group level. Instead, our cohort predominantly exhibited a sensory loss-of-function phenotype over the neck and upper extremities. The neuropathic pain descriptors in the upper quadrant reported by a subset of follow-up WADII participants (e.g. burning, tingling) differ from the diffuse, deep and achy characteristics typically associated with nociplastic pain.^57^ Importantly, the nociplastic pain grading system necessitates the exclusion of neuropathic pain before a diagnosis of nociplastic pain can be established.^58^ Although neurological loss-of-function has been observed in non-neuropathic conditions,^59,60^ the limited evidence of local and widespread hypersensitivity in our cohort, coupled with longitudinal findings that demonstrate an overall trend towards improvement, does not suggest clear nociplastic pain phenotypes at the group level.

The lack of group-level signs of central sensitization in our study is likely to reflect the inclusion of both recovered and non-recovered participants with WADII, in addition to our focus on characterizing neurological loss-of-function and neuropathic pain within the upper quadrant. The inclusion of pain data from a full-body chart and additional gain-of-function measures over the leg (e.g. thermal pain thresholds or temporal summation in addition to pressure pain thresholds) might have provided greater clarity on the presence or absence of widespread hypersensitivities in our cohort. Our 6-month follow-up time point might also be too early to capture clear nociplastic presentations and indications of central sensitization, which are more commonly observed at later stages in patients who do not recover.^56^

### Structural parameters indicate reduced dermal, but not intraepidermal, nerve fibres 6 months post-injury

At 6 months, skin biopsy analyses suggest a reduction in dermal nerve bundles, with the preservation of intraepidermal nerve fibres. This reduction might be one explanation for the continued large-fibre sensory hypoaesthesia identified in a subset of corresponding follow-up participants. However, this was a secondary analysis with a limited sample size (*n* = 30), requiring replication in independent cohorts.

In a previous cross-sectional study in chronic WADII, no changes in Meissner corpuscles or myelinated dermal bundles at the index finger were reported. However, this study identified a reduction in intraepidermal nerve fibre densities and dermal nerve bundles at the index finger.^13^ The divergence in some of the findings is likely to stem from the clear differences between cohorts; the previous study focused on a smaller cohort of participants with WADII, all of whom had persistent pain well beyond 6 months (median duration 5 years).^13^ It is therefore possible that the previously identified long-term reduction in intraepidermal nerve fibre densities is ascribed to secondary mechanisms (e.g. systemic inflammation,^61^ reduced activities levels^62^) rather than directly related to the injury.

### Acute sensory hypoaesthesia persists in a subgroup at 6 months and predicts persistent upper quadrant pain

Our longitudinal assessment of sensory function reveals mixed patterns of persistent hypoaesthesia in some individuals following whiplash injury. Clinical neurological assessment showed a reduction in hypoaesthesia from 54% acutely to 25% at 6 months. Remarkably, the highly sensitive QST results suggest persistent hypoaesthesia over the index finger in a large proportion of participants at follow-up, with 67.6% affected acutely and 74.6% at 6 months. This discrepancy highlights the potential limitations of current clinical tools to detect subtle somatosensory changes.^63^ The only other study examining a limited battery of temporal loss-of-function from acute onset WADII supports our findings.^64^ Here, we included much more detailed sensory profiling, revealing that somatosensory hypoaesthesia might even expand over time for a subset of participants with WADII, including to the leg.

Our data suggest that sensory hypoaesthesia measured with low-cost bedside tests is not only important for evaluating neurological function over time but might also be prognostically relevant for whiplash-related upper quadrant pain 6 months post-injury in WADII. Previous systematic reviews suggest a preliminary negative association between recovery and the presence of frank nerve lesions (WAD grade III).^5,47^ The prognostic value of hypoaesthesia was evidenced by use of clinically available and low-cost tests and might have been overlooked in previous research focused primarily on neurological gain-of-function. Future studies should consider sensory hypoaesthesia when validating large prognostic models.

### Clinical implications of nerve pathology in WADII

Our findings challenge the view that WADII is purely musculoskeletal and emphasize the importance of including comprehensive neurological assessments. Our detailed clinical neurological assessment revealed that sensory hypoaesthesia was determined predominantly by the function of small nerve fibres, suggesting the need to perform a comprehensive neurological assessment, including pinprick and temperature. Such sensory examination not only informs the current whiplash grading,^4^ but is also an essential part of determining the presence of neuropathic pain.^29^ Sensory hypoaesthesia at the index finger might also have prognostic value for persistent upper quadrant pain, although further validation is required.

Early identification of nerve pathology and neuropathic pain represents a potential opportunity to target timely interventions and personalized treatment approaches for neuropathic pain in the most common type of whiplash injury. Accurately classifying neuropathic pain might help to validate and explain patients’ symptoms and enable the initiation of specific physiotherapeutic^65,66^ or pharmacological^67^ interventions. Despite the high prevalence of neuropathic pain identified in our cohort, a very small minority of participants received targeted neuropathic pain treatment (e.g. 0.8% receiving first-line neuropathic pain medication), which is, potentially, a missed opportunity. Recent studies suggest that first-line neuropathic pain medications are feasible and well tolerated in WAD,^68,69^ with large-scale randomized controlled trials underway to evaluate their efficacy in managing WADII symptoms and prevention of pain persistence.

### Limitations

A key strength of our study is the longitudinal, multi-modal phenotyping, starting in the acute phase after whiplash injury. However, there are limitations to consider. Our numbers recruited (*n* = 62 acute WADII and *n* = 36 controls) were slightly less than the target sample size for the skin biopsy analysis (*n* = 68 acute WADII and *n* = 36 control skin biopsies). However, this slight shortfall is unlikely to have influenced our results greatly. Although we deliberately took a pragmatic approach to the clinical examination, which enabled integration of information on symptoms and signs, practical constraints prevented us from blinding the examiners to the status of participant. However, we adhered strictly to the validated QST protocol, including standardized wording to minimize examiner bias.

Given that all participants with WADII were recruited from accident and emergency departments, the generalizability of our results might be limited. Although attendance in emergency departments acutely after whiplash injuries is common,^70,71^ some patients might access the health service via different pathways. Although the multicentre nature of this study improves generalizability, it creates potential for variance in outcomes between centres. We used several strategies to mitigate inter-rater effects, including selection of valid and reliable clinical assessments, harmonization and pilot testing among assessors, and investigation of inter-rater reliability of intraepidermal nerve fibre counts.

## Conclusion

Our study shows conclusively that a significant proportion of participants with WADII exhibit signs of neuropathic pain and nerve pathology in the acute phase after whiplash injury. Although most participants showed signs of recovery, a subgroup of participants with WADII experienced persistent neuropathic symptoms and sensory hypoaesthesia 6 months post-injury. Of note, acute hypoaesthesia measured with low-cost tools at the bedside was prognostic for whiplash-related upper quadrant pain at 6 months.

Our study provides novel contributions to the understanding of WADII pathophysiology, emphasizing a spectrum of nerve involvement and complex clinical phenotypes. Our findings have important clinical implications, underscoring the need for comprehensive neurological assessments and monitoring in WADII. Future research is needed to investigate the underlying mechanisms driving the diverse neurological phenotypes and to evaluate the efficacy of targeted interventions for the subgroup of participants with WADII exhibiting signs of neuropathic pain.

## Supplementary Material

awaf088_Supplementary_Data

## Data Availability

The anonymized data from this study is available from the Oxford University Research Archive at http://dx.doi.org/10.5287/ora-ye0n8mdgv.
