# Supplementary material for: The presence and prognosis of nerve pathology following whiplash injury: a prospective cohort study
Source: Brain. 2025 Mar 5;148(9):3392–406. doi: 10.1093/brain/awaf088 (PMC12404775; doi:10.1093/brain/awaf088)
Supplement: awaf088_Supplementary_Data [file awaf088_supplementary_data.zip › brain-2024-02337-File011.pdf]

Supplementary Material

SUPPLEMENTARY METHODS

Participant Characteristics

Healthy control cohort

In addition to newly recruited healthy control participants, additional age- and sex-matched controls were drawn from three cohorts recruited at the University of Oxford (The Oxford Carpal Tunnel Syndrome Cohort[1,2], FORECAST[3], and MONET[4]) including 37 additional participants for QST, 18 for skin biopsies, and 11 for serum NfL. QST z-scores were calculated per age decade using at least n = 7 healthy controls per decade[5] (totalling N=79 separate controls in the final QST analysis). Similar selection criteria were used for healthy control participants from all studies[1-4,6]. A summary of healthy control participants used in each analysis is included in Supplementary Figure 1.

For accurate comparison of intraepidermal nerve fibre densities between the whiplash and Oxford Carpal Tunnel Syndrome Cohort[1,2], two assessors (AS and JF) independently counted the same nine sections of stained skin samples, demonstrating excellent inter-rater reliability (ICC = 0.92, 95% CI= 0.70, 0.99).

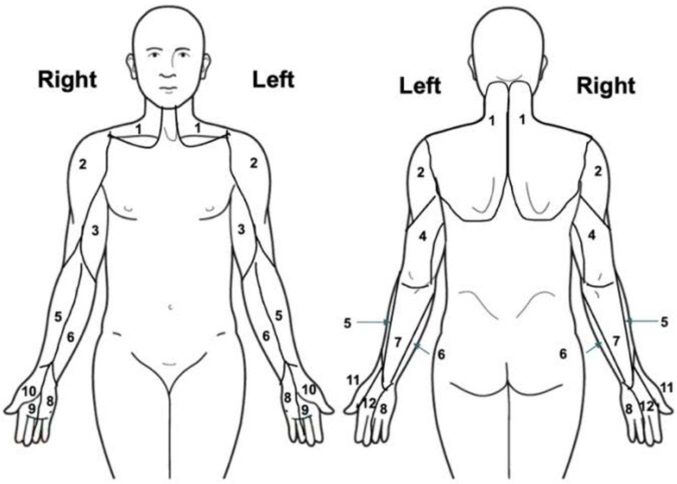

Supplementary Figure 1. Pain body diagram for upper quandrant pain, including the neck and upper extremities.

Bedside neurological assessment

Testing procedures:

A summary of the site and grading criteria for the bedside neurological assessments is included in Supplementary Table 1. All bedside neurological assessments were tested in WADII and healthy control participants bilaterally.

Supplementary Table 1. Summary of clinical neurological assessments and corresponding grading scales.

Site	Test	Grading		
		**Loss of function**	**Normal**	**Gain of function**
**Innervation territories (C5-T1)**	Muscle strength	0: No contraction, 1: Flicker or trace of contraction, 2: Active movement, with gravity eliminated, 3: Active movement against gravity, 4: Active movement against gravity and resistance	5: Normal power.	NA
	Deep tendon reflexes	0: no response, 1: slight response	2: brisk, normal response;	3: very brisk response.
	Cutaneous sensation (cotton wool, pinprick, thermal coins)	0: Absent, 1: Reduced	2: Normal;	3: Enhanced
**Main pain area**	Cutaneous sensation (pinprick & thermal coins)	0: Absent, 1: Reduced	2: Normal;	3: Enhanced.

**Index finger**	Cutaneous sensation (pinprick & thermal coins)	0: Absent, 1: Reduced;	2: Normal	3: Enhanced.

Muscle strength: Myotomal isometric muscle strength upon manual testing was graded using the six-point ordinal MRC grading scale (0: no contraction, 5: normal power[7]). Tested muscles included: C5: deltoid, C6: biceps brachii, C7: triceps brachii, C8: extensor pollicis longus and abductor pollicis, T1: dorsal and palmar interossei.

Deep tendon reflexes: the biceps and triceps brachii muscles were tested in the upper extremities using a four-point ordinal scale (0: absent, 3: very brisk response).

Cutaneous sensation: Light touch (cotton wool), and pinprick (using neurotips) sensation testing was performed over the C5-T1 innervation territories. In addition, pinprick and thermal sensation (using warm and cool coins) was assessed at the index finger and self-reported main pain area. For thermal testing, two metal coins (50 pence pieces) were used. One coin was left at room temperature (cool coin) and one coin was left in the pocket of the researcher for at least 30 minutes (warm coin), as previously described and validated[8]. Each test was first performed on the side contralateral to the most symptomatic upper extremity. Participants were asked to compare the perceived sensation on the most symptomatic side to the initially tested less symptomatic side. If a participant reported a sensation as abnormal, the test was repeated a maximum of two times to confirm the initially reported finding. If participants reported equal symptom laterality, the pectoralis muscle was used as a normative area. All cutaneous sensation was measured using a four-point ordinal scale (0: absent; 1: reduced; 2: normal; 3: enhanced)[9].

Interpretation of neurological bedside assessments

Neurological loss of function was classified for each corresponding test according to the following criteria: cutaneous loss of function was considered if participants subjectively reported reduced or absent cutaneous sensation to cotton wool, pinprick, thermal coins; isometric muscle strength values less than five on the MRC grading scale[7]; biceps or triceps deep tendon reflex values less than two (normal, brisk response) out of four[10].

Neurological gain of function was categorised for each corresponding test according to the following criteria: subjectively reported enhanced cutaneous sensation to light touch (e.g., reports of paraesthesia or allodynia) or pinprick (e.g., increased painful sensation); biceps or triceps deep tendon reflex values greater than two out of four[10].

‘Mixed’ classification for bedside neurological assessments had to include at least one test classified as loss of function and another test classified as gain of function.

Psychometric properties

Clinical assessments of neurological function, including reflexes, myotomal strength, and cutaneous light touch sensation have previously been shown to have moderate to substantial reliability[11]. The pinprick and thermal coin testing have been shown to be a valid method to detect somatosensory dysfunction[12] and small-fibre degeneration[8], including in the upper limb.

Standardisation across sites:

All clinical assessments were piloted and standardised among the clinical researchers at Oxford University (SK, JF) and the Brighton and Sussex Medical School (CR). Both sites used identical testing equipment.

Quantitative sensory testing (QST)

Analysis: Data transformation and analysis for all QST parameters followed previously recommended methods for clinical testing[13,14]. All testing parameters, excluding CPT, HPT, and VDT, were log transformed to achieve normal distribution. MPS values included the addition of a constant 0.1 to avoid losing zero-rated values. A lack of variance for VDT due to ceiling effects within the 20-year-old age decade did not allow for z-score calculation. Therefore, VDT z-scores for 20- and 30-year-olds were derived from a combined total mean and standard deviation incorporating both decades into one calculation. The remaining VDT z-scores were calculated for each age decade.

Sample size estimations

To comprehensively assess the role of nerve pathology in WADII, we designed our study to evaluate both the presence and prognostic ability of several factors. These factors include structural signs of nerve pathology using immunohistochemistry of skin biopsies and serum levels of neurofilament light chain (NfL). All sample size calculations were performed using G*Power (version 3.1.9.6)[15].

Determining the presence of structural nerve fibre pathology during the acute and chronic stages using skin biopsies: 68 WADII participants and 34 healthy control participants (80% power, 5% significance, 0.53 effect size) would allow the detection of a 20% smaller difference in intraepidermal nerve fibre density in the acute stage compared to healthy controls based on our previous cohort of participants with chronic WADII[16].

Assessing the presence and temporal profile of axonal injury using a serum measure of nerve pathology (NfL). Based on a previous study analysing plasma neurofilament light chain concentrations in participants after acute non-brain injury trauma compared to healthy controls,[17] n = 15 participants are required in each group to detect a significant difference ($d = 1.4$, power = 0.95. $\alpha = 0.05$) using a two-sided independent t-test.

Revealing whether signs of acute nerve dysfunction predict neck-related disability at 6-months. A sample size of 69 WADII participants provides 80% power to detect a squared partial correlation of 13% between 6-month disability scores (NDI) and NfL measures (mean concentration and z-scores). This corresponds to a medium effect size $f^2=0.15$, in a linear regression model at a 5% significance level, controlling for the known factors age, sex and initial NDI allowing for 20% drop-out (N = 55 paired WADII participants required for final analysis).

SUPPLEMENTARY RESULTS

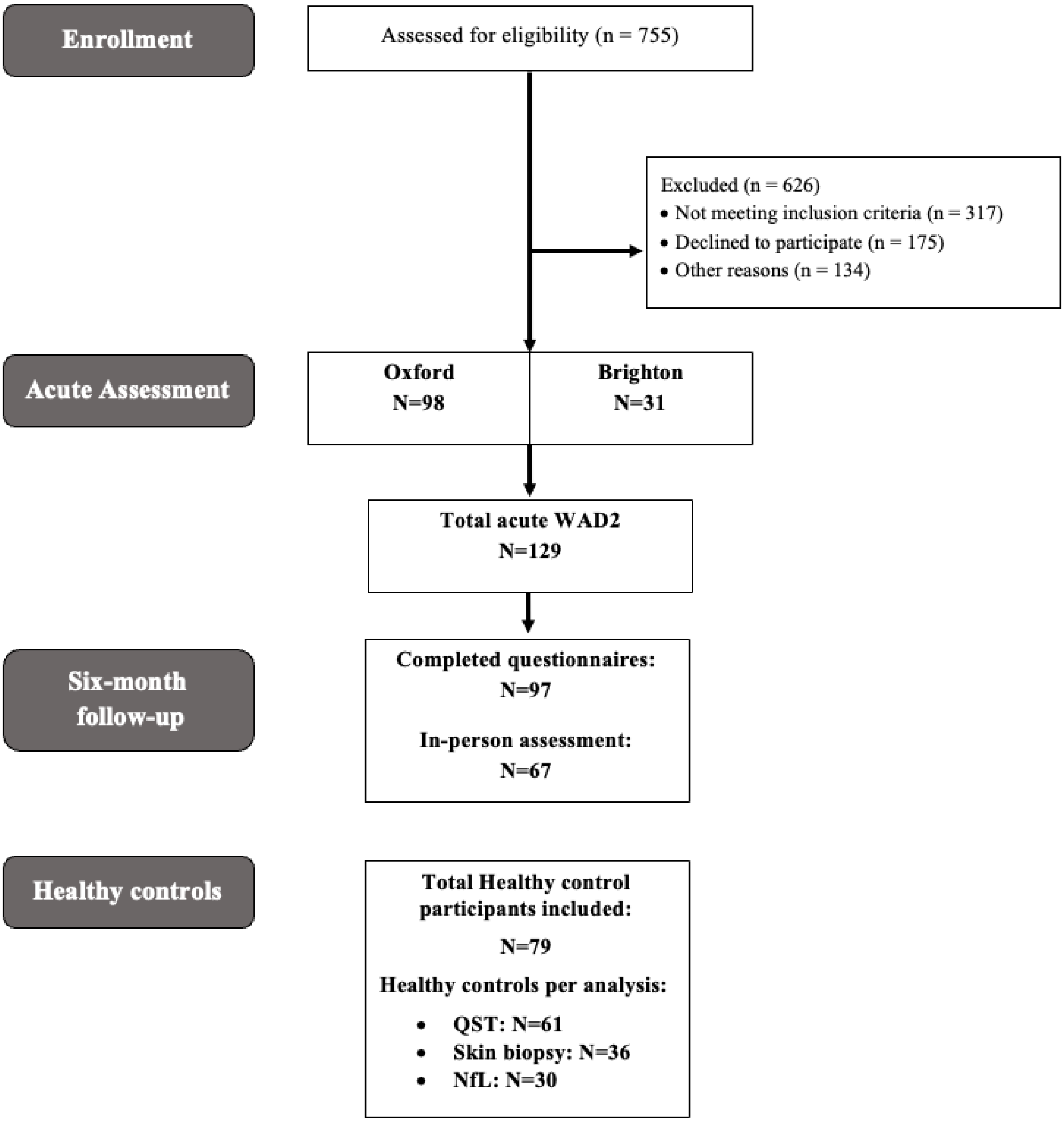

Supplementary Figure 2. Study flow diagram.

WADII cohort characteristics

Supplementary Table 2. Additional acute and follow-up WADII participant characteristics.

	**Acute**	**Follow-up**	**P-value**
**Number of participants**	129	97 completed questionnaires<br><br>67 attended in-person assessment	
**Time of acute assessment (days post-injury, median/IQR)**	24 (13)	NA	
**DASS – depression (med/IQR)**	6.0 (11.5)<br>N=123	3.0 (10.3)<br>N=92	**0.02**
**DASS – anxiety (med/IQR)**	6.0 (13.4)	4.0 (8.0)	**0.004**
**DASS – stress (med/IQR)**	12.0 (12.0)	9.5 (13.5)	**0.01**
**EQ-5D-5L (Index value; mean/SD)**	0.73 (0.2)<br>N=121	0.83 (0.1)<br>N=93	**< 0.001**
**PTSD-8 total (med/IQR)**<br>Likely PTSD	18.0 (9.0)<br>51% (62/121)	14.0 (11.0)<br>37% (34/91)	**< 0.01**
**Impact of Events Scale – Revised (med/IQR)**<br>Probable PTSD	27.0 (28.0)<br>40% (43/107)	15.0 (27.5)<br>24% (21/88)	**< 0.001**
**PCS (med/IQR)**<br>Likely catastrophizing (n/%)	13.0 (17.0)<br>13% (15/116)	7.5 (13.0)<br>3% (3/92)	**< 0.001**
**Recovery question**			
All better	NA	26% (25/97)	
Quite a bit of improvement		45% (44/97)	
Some improvement		22% (21/97)	
No improvement		2% (2/97)	
A little worse		4% (4/97)	
Much worse		1% (1/97)	
**Medications related to whiplash injury**			
Paracetamol	74% (96/129)	24% (16/67)	
NSAIDs	59% (76/129)	10% (7/67)	
Weak opioids	57 % (73/129)	-	
Others:	33 % (42/129)	-	
Diazepam	3% (4/129)	-	
Deep heat (patch, rub)	1% (2/129)	1% (1/67)	
Diclofenac	0.8% (1/129)	-	
Amitriptyline	0.8% (1/129)	-	
Etoricoxib	0.8% (1/129)	-	
Amitriptyline	-	1% (1/67)	
Citalopram	-	1% (1/67)	

Numerical data are presented as median (IQR) when non-parametric, mean (SD) when normally distributed, or percentage (%) for categorical data PTSD-8 scoring: likely PTSD (≥18/32). Impact of Events Scale scoring: probable PTSD (≥33/88). PCS >30 indicates significant level of pain catastrophising. Healthy control participants did not complete self-reported outcome measures and, by definition of inclusion criteria, did not have any neck or arm symptoms. Wilcoxon signed-rank tests were used for all non-parametric comparisons and t-test was used for normally distributed variables (EQ-5D-5L index value). Weak opioids included: tramadol, codeine, co-codamol, co-dydramol, or co-proxamol. Bolded text indicated statistical significance (set at $p < 0.05$).
Abbreviations: BMI: body mass index; DASS: Depression Anxiety and Stress Scale; IQR: interquartile range; med: median; PTSD: posttraumatic stress disorder; PCS: Pain Catastrophizing Scale MRI: magnetic resonance imaging; CT: computerized tomography, NSAID: nonsteroidal anti-inflammatory, TENS: Transcutaneous electrical nerve stimulation.

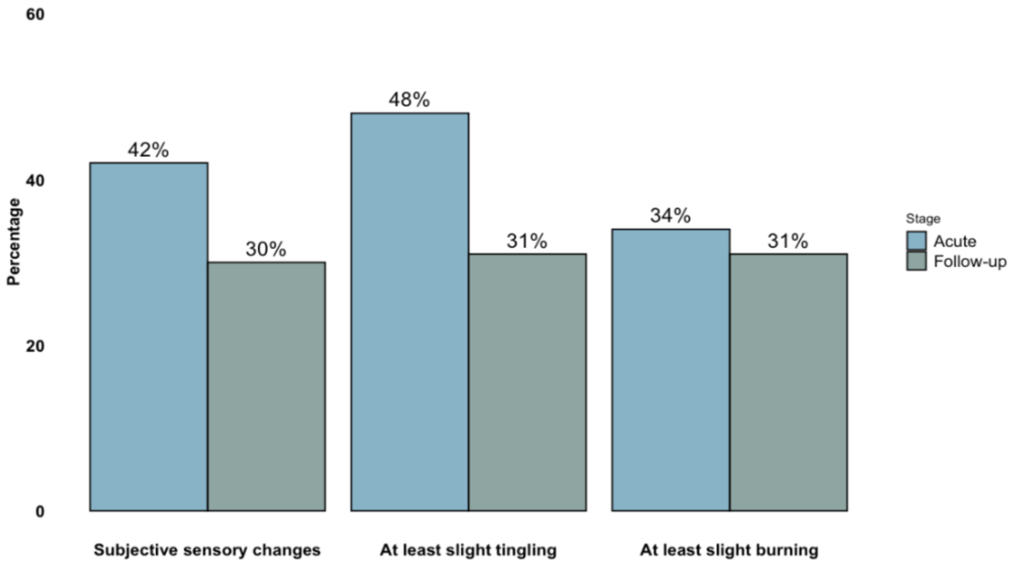

Supplemental Figure 3. Reported neuropathic pain characteristics, including subjective reports of sensory changes (e.g., numbness, tingling); burning and tingling measures were taken from the corresponding questions of the painDETECT questionnaire.

Bedside neurological assessment

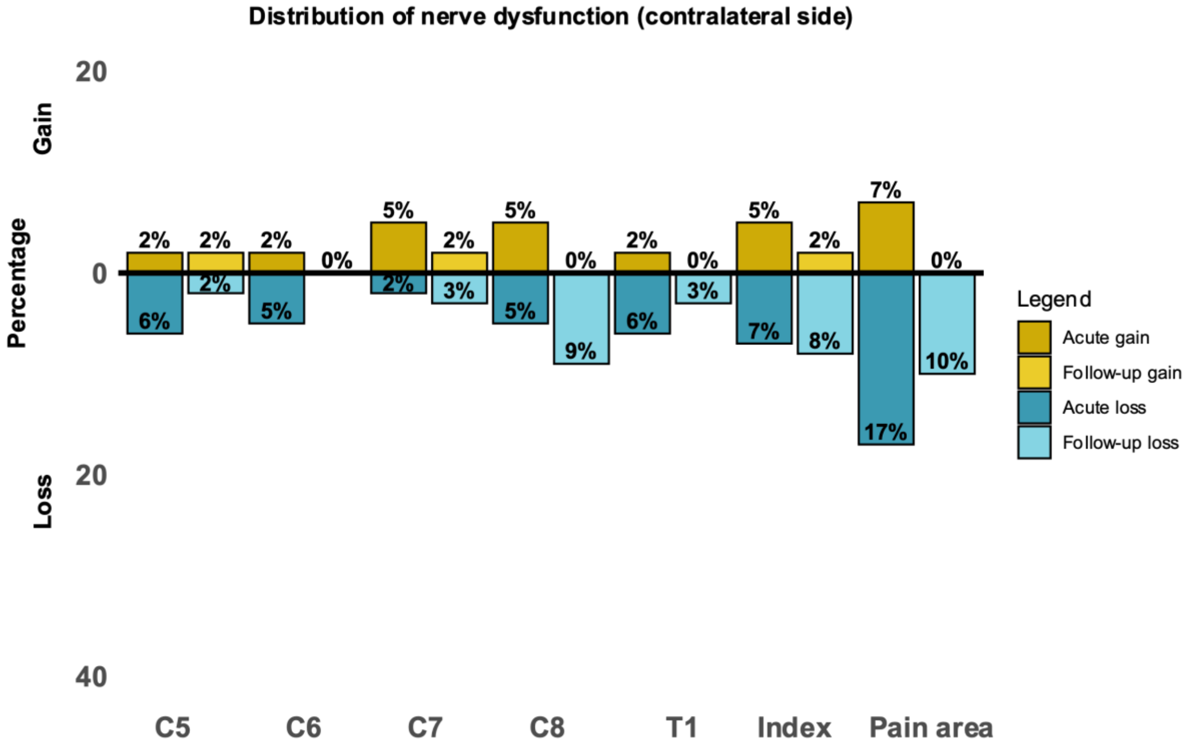

Supplemental Figure 4. Bedside neurological assessment on the contralateral side identifies a less pronounced loss of function phenotype throughout the upper extremity that persists at follow-up. (A) Percentage of composite neurological dysfunction in the side contralateral to the most symptomatic C5-T1 innervation territories, index finger, and main pain area for acute and follow-up, including strength, reflexes, light touch, pinprick, and thermal coins. N=129 acute and n=67 follow-up participants.

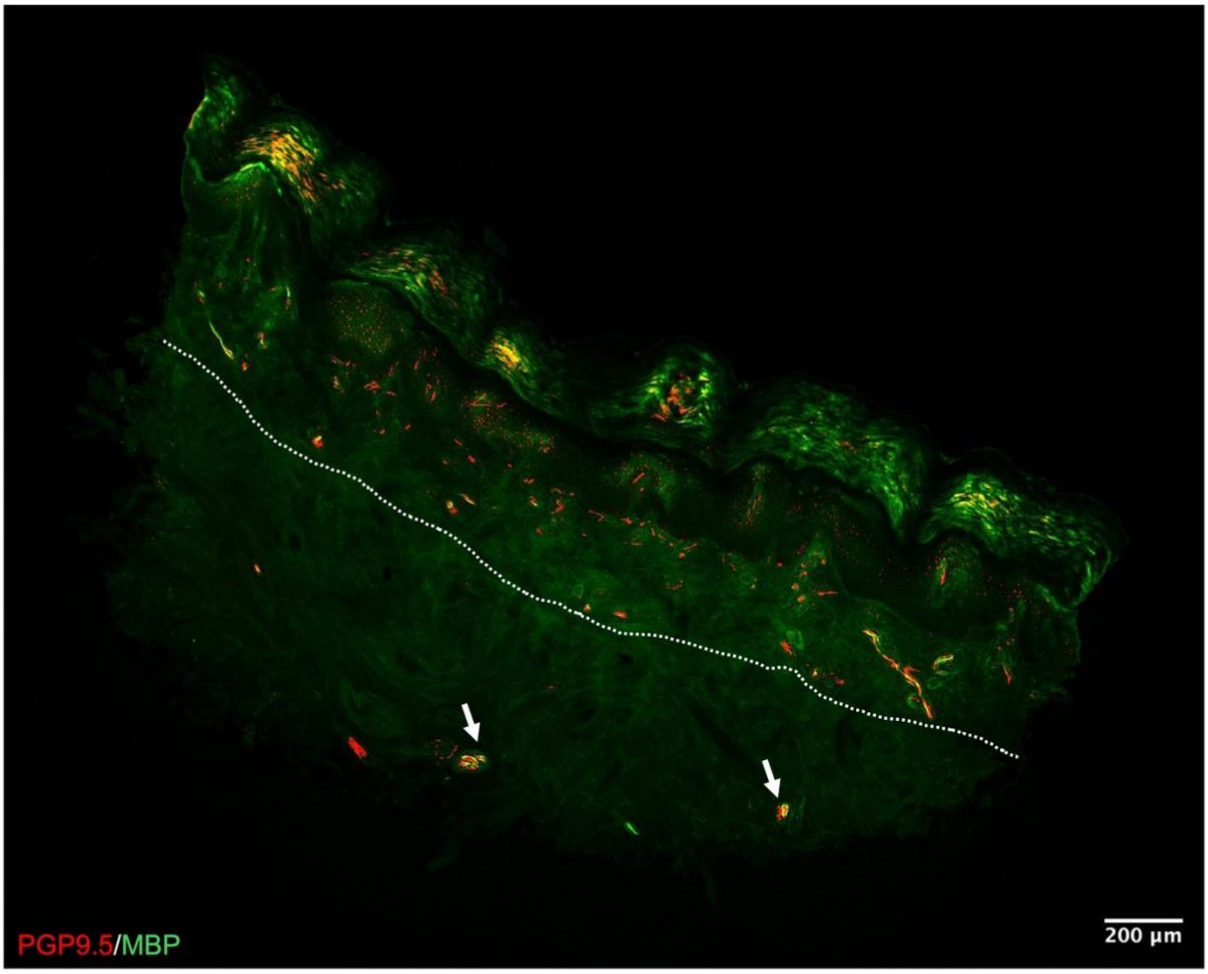

Supplemental Figure 5. Example image of a human skin demonstrating dermal nerve bundles in an acute WADII participant. Dermal bundles were counted in the lower dermis (dotted area), excluding the subepidermal plexus. The white arrows indicate dermal bundles with at least five PGP9.5+ axons (red) containing MBP (green).

Abbreviations: MBP: myelin basic protein, PGP: protein gene product.

Supplementary Table 3. Means and standard deviations for QST z-scores by group

Variable	Acute WADII	Follow-up WADII
mean_CDT	-2.02	-1.14
sd_CDT	3.99	2.15
mean_WDT	-0.99	-0.70
sd_WDT	1.70	1.66
mean_TSL	-0.96	-0.82
sd_TSL	1.31	1.12
mean_CPT	0.22	0.36
sd_CPT	1.14	1.10
mean_HPT	-0.07	0.10
sd_HPT	1.01	1.00
mean_MDT	-1.37	-1.23
sd_MDT	1.75	1.70
mean_MPT	0.35	0.01
sd_MPT	1.54	1.32
mean_MPS	0.14	-0.45
sd_MPS	1.49	0.89
mean_WUR	0.11	0.02
sd_WUR	0.98	1.05
mean_VDT	-4.19	-0.96
sd_VDT	12.01	4.84
mean_PPT	-0.17	-0.98
sd_PPT	1.71	1.34
mean_CDT_leg	-1.34	-1.74
sd_CDT_leg	4.48	5.70
mean_WDT_leg	-0.40	-0.69
sd_WDT_leg	1.41	1.61
mean_PPT_leg	-0.004	-0.35
sd_PPT_leg	1.49	1.34

Abbreviations: CDT: cold detection threshold; CPT: cold pain threshold; HPT: heat pain threshold; MDT: mechanical detection threshold; MPS: mechanical pain sensitivity; MPT: mechanical pain threshold; PPT: pressure pain threshold; TSL: thermal sensory limen; VDT: vibration detection threshold; WDT: warm detection threshold; WUR: wind-up ratio; Sd: standard deviation.

Supplementary Table 4. One-way ANOVA results assessing QST parameters between controls, acute WADII, and follow-up WADII

	Acute vs follow-up	Acute vs HC	Follow-up vs HC
**CDT**	0.14	**<0.001**	0.09
**WDT**	0.43	**<0.001**	**0.04**
**TSL**	0.72	**<0.001**	**<0.001**
**CPT**	0.68	0.39	0.15
**HPT**	0.47	0.88	0.83
**MDT**	0.83	**<0.001**	**<0.001**
**MPT**	0.24	0.23	0.99
**MPS**	**<0.01**	0.99	**0.02**
**WUR**	0.82	0.61	0.95
**VDT**	**0.04**	**<0.01**	0.81
**PPT**	**<0.001**	0.75	**<0.001**
**CDT – leg**	0.84	0.21	0.12
**WDT – leg**	0.37	0.30	**0.04**
**PPT – leg**	0.22	0.99	0.40

Data are presented as adjusted p-values from a one-way ANOVA with Tukey's Honest Significant Difference post hoc testing. Bolded text indicates statistical significance (set at $p < 0.05$).

Abbreviations: CDT: cold detection threshold; CPT: cold pain threshold; HPT: heat pain threshold; MDT: mechanical detection threshold; MPS: mechanical pain sensitivity; MPT: mechanical pain threshold; PPT: pressure pain threshold; TSL: thermal sensory limen; VDT: vibration detection threshold; WDT: warm detection threshold; WUR: wind-up ratio.

Measures of structural nerve pathology in WADII

Supplementary Table 5. Intraepidermal nerve fibre densities and dermal fibres at the index finger and distal leg.

	Acute WADII	Follow-up WADII	Controls	F	Sig.	Acute v control	Acute v follow-up	Follow-up v control
**Index finger**								
IENFD med/IQR (fibres/mm)	9.9 (6.1)	11.3 (11.8)	8.7 (3.8)	2.3	P = 0.11	P = 0.39	P = 0.50	P = 0.09
Meissner's corpuscles med/IQR, (number/mm)	0.62 (0.7)	0.56 (0.5)	0.64 (0.6)	0.2	P = 0.82	P = 0.84	P = 0.99	P = 0.86
PGP+/MBP bundles	2.9 (2.3)	2.5 (2.9)	4 (2.8)	4.1	**P = 0.02**	P = 0.08	P = 0.61	**P = 0.02**
**Distal leg**								
IENFD med/IQR (fibres/mm)	8.1 (6.4)	10.4 (6.1)	8.8 (6.2)	0.64	P = 0.53	P = 0.92	P = 0.50	P = 0.79
PGP+/MBP bundles	0.4 (0.8)	0.4 (0.7)	0.3 (0.9)	0.09	P = 0.91	P = 0.95	P = 0.92	P = 0.99

Index finger skin biopsies were taken from the volar aspect of the proximal phalanx of the index finger for all participants. Index finger skin biopsies for acute WADII was taken from the most symptomatic side and from the non-dominant side for controls. Aligned ranks transformation ANOVA with Tukey's post hoc testing was used for all comparisons. Index finger data includes N=62 acute WADII, N=30 follow-up WADII, and N=38 controls. Distal leg data include N=40 acute WADII, N=23 follow-up WADII, and N=22 controls.
Abbreviations: IENFD: intraepidermal nerve fibre density; IQR: interquartile range; Med: median.

Supplementary Table 6. Acutely elevated serologic NfL levels return to baseline at six-months

	Controls	Acute WADII	Follow-up WADII	F	Sig.	Acute v control	Acute v follow-up	Follow-up v control
**Serum NfL concentration (pg/mL)** median (IQR)	5.24 (3.5)	7.13 (5.3)	5.61 (4.6)	3.62	**P = 0.03**	**P = 0.04**	P = 0.20	P = 0.69
**Serum NfL z-scores** (mean/SD)	-0.766 (1.2)	0.016 (1.4)	-0.55 (1.1)	5.58	**P = 0.005**	**P = 0.01**	P = 0.05	P = 0.75

Aligned ranks transformation ANOVA with Tukey's post hoc testing was used to compare absolute serum NfL concentrations. One-way ANOVA with Tukey's Honest Significant Difference post hoc testing was used to compare NfL z-scores. Bolded text indicates statistical significance (set at $p < 0.05$). N=30 controls, N=91 acute WADII, N=41 follow-up WADII were included for analysis.
Abbreviations: NfL: Neurofilament light chain; SD: standard deviation; IQR: interquartile range. F: F-statistic of one-way ANOVA; Sig: p-value for overall one-way ANOVA analysis.

Supplementary Table 7. Summary of effect sizes (95% confidence intervals) for measures of nerve pathology.

Variable	Control vs Acute	Acute vs follow-up	Control vs follow-up
CDT	0.66 (0.35, 0.97)	-0.29 (-0.59, 0.01)	0.37 (0.02 0.72)
WDT	0.62 (0.31, 0.94)	-0.19 (-0.49, 0.11)	0.44 (0.09, 0.79)
TSL	0.81 (0.49, 1.12)	-0.12 (-0.42, 0.18)	0.69 (0.34, 1.04)
CPT	-0.20 (-0.51, 0.10)	-0.13 (-0.43, 0.17)	-0.33 (-0.68, 0.02)
HPT	0.07 (-0.23, 0.38)	-0.18 (-0.48, 0.12)	-0.10 (-0.45, 0.25)
MDT	0.87 (0.55, 1.19)	-0.09 (-0.39, 0.21)	0.78 (0.43, 1.14)
MPT	-0.25 (-0.56, 0.05)	0.24 (-0.05, 0.54)	-0.01 (-0.36, 0.34)
MPS	-0.001 (-0.31, 0.31)	0.47 (0.17, 0.77)	0.47 (0.12, 0.82)
WUR	-0.15 (-0.46, 0.16)	0.09 (-0.21, 0.40)	-0.06 (-0.41, 0.30)
VDT	0.47 (0.16, 0.79)	-0.37 (-0.67, -0.06)	0.11 (-0.24, 0.46)
PPT	0.11 (-0.19, 0.42)	0.55 (0.25, 0.86)	0.67 (0.31, 1.02)
CDT – leg	0.29 (-0.05, 0.64)	0.09 (-0.22, 0.40)	0.38 (-0.001, 0.77)
WDT – leg	0.26 (-0.09, 0.60)	0.21 (-0.10, 0.53)	0.47 (0.08, 0.86)
PPT – leg	-0.01 (-0.35, 0.34)	0.26 (-0.05, 0.56)	0.25 (-0.13, 0.63)
Index finger intraepidermal nerve fibre density	-0.27 (-0.69, 0.14)	-0.25 (-0.67, 0.16)	-0.53 (-0.95, -0.11)
Index finger Meissner corpuscle	-0.12 (-0.53, 0.30)	-0.01 (-0.43, 0.40)	-0.13 (-0.55, 0.29)
Index finger MBP+/PGP+ bundles/mm^2	0.47 (0.05, 0.89)	0.21 (-0.20, 0.63)	0.69 (0.26, 1.11)
Distal leg intraepidermal nerve fibre density	0.10 (-0.43, 0.63)	-0.30 (-0.83, 0.24)	-0.20 (-0.73, 0.34)
Distal leg MBP+/PGP+ bundles/mm^2	-0.08 (-0.61, 0.45)	0.10 (-0.43, 0.63)	0.02 (-0.51, 0.55)
NfL concentration (pg/mL)	-0.52 (-0.94, -0.10)	0.32 (-0.10, 0.74)	-0.20 (-0.61, 0.22)
NfL z-scores	0.62 (0.20, 1.04)	0.44 (0.07, 0.82)	0.17 (-0.65, 0.30)

Effect size data are presented as Cohen's d (95% confidence intervals).

Abbreviations: CDT: cold detection threshold; CPT: cold pain threshold; HPT: heat pain threshold; MDT: mechanical detection threshold; MPS: mechanical pain sensitivity; MPT: mechanical pain threshold; NfL: neurofilament light chain; PPT: pressure pain threshold; TSL: thermal sensory limen; VDT: vibration detection threshold; WDT: warm detection threshold; WUR: wind-up ratio; Sd: standard deviation.

References:

1. Baskozos G, Sandy-Hindmarch O, Clark AJ, *et al*. Molecular and cellular correlates of human nerve regeneration: ADCYAP1/PACAP enhance nerve outgrowth. *Brain*. Jul 1 2020;143(7):2009-2026. doi:10.1093/brain/awaa163
2. Schmid AB, Bland JD, Bhat MA, Bennett DL. The relationship of nerve fibre pathology to sensory function in entrapment neuropathy. *Brain*. Dec 2014;137(Pt 12):3186-99. doi:10.1093/brain/awu288
3. Schmid AB, Ridgway L, Hailey L, *et al*. Factors predicting the transition from acute to persistent pain in people with 'sciatica': the FORECAST longitudinal prognostic factor cohort study protocol. *BMJ Open*. 2023;13(4):e072832. doi:10.1136/bmjopen-2023-072832
4. E S-S, M T, Ac T, M S, G B, Ab S. Mechanisms of neurodynamic treatments (MONET): a protocol for a mechanistic, randomised, single-blind controlled trial in patients with carpal tunnel syndrome. *BMC Musculoskeletal Disorders*. 2024/07/27 2024;25(1):590. doi:10.1186/s12891-024-07713-6
5. Blankenburg M, Boekens H, Hechler T, *et al*. Reference values for quantitative sensory testing in children and adolescents: developmental and gender differences of somatosensory perception. *Pain*. Apr 2010;149(1):76-88. doi:10.1016/j.pain.2010.01.011
6. Ridehalgh C, Fundaun J, Bremner S, *et al*. Does peripheral neuroinflammation predict chronicity following whiplash injury? Protocol for a prospective cohort study. *BMJ Open*. 2022;12(12):e066021. doi:10.1136/bmjopen-2022-066021
7. Memorandum M. *Aids to the examination of the peripheral nervous system*. HMSO, London; 1976.
8. Ridehalgh C, Sandy-Hindmarch OP, Schmid AB. Validity of Clinical Small-Fiber Sensory Testing to Detect Small–Nerve Fiber Degeneration. *Journal of Orthopaedic & Sports Physical Therapy*. 2018;48(10):767-774. doi:10.2519/jospt.2018.8230
9. Ropper AH, RH B. *Adams and Victor's Principles of Neurology 8th Edition*. McGraw-Hill, Medical Publishing Division; 2005.
10. Hallett M. NINDS Myotatic Reflex Scale. *Neurology*. 1993;43(12):2723-2723. doi:10.1212/wnl.43.12.2723
11. B. SA, Brunner F, Luomajoki H, *et al*. Reliability of clinical tests to evaluate nerve function and mechanosensitivity of the upper limb peripheral nervous system. *BMC musculoskeletal disorders*. 01/21/2009 2009;10doi:10.1186/1471-2474-10-11
12. Zhu GC, Böttger K, Slater H, *et al*. Concurrent validity of a low-cost and time-efficient clinical sensory test battery to evaluate somatosensory dysfunction. *European journal of pain (London, England)*. 2019;23(10):1826-1838. doi:10.1002/ejp.1456
13. Rolke R, Magerl W, Campbell KA, *et al*. Quantitative sensory testing: a comprehensive protocol for clinical trials. *Eur J Pain*. Jan 2006;10(1):77-88. doi:10.1016/j.ejpain.2005.02.003
14. Rolke R, Baron R, Maier C, *et al*. Quantitative sensory testing in the German Research Network on Neuropathic Pain (DFNS): standardized protocol and reference values. *Pain*. Aug 2006;123(3):231-243. doi:10.1016/j.pain.2006.01.041
15. Faul F, Erdfelder E, Lang AG, Buchner A. G*Power 3: a flexible statistical power analysis program for the social, behavioral, and biomedical sciences. *Behav Res Methods*. May 2007;39(2):175-91. doi:10.3758/bf03193146
16. Farrell SF, Sterling M, Irving-Rodgers H, Schmid AB, Irving-Rodgers H. Small fibre pathology in chronic whiplash-associated disorder: A cross-sectional study. *Eur J Pain*. 2020;24(6):1045-1057. doi:10.1002/ejp.1549
17. Graham NSN, Zimmerman KA, Moro F, *et al*. Axonal marker neurofilament light predicts long-term outcomes and progressive neurodegeneration after traumatic brain injury. *Sci Transl Med*. Sep 29 2021;13(613):eabg9922. doi:10.1126/scitranslmed.abg9922
